# Genome-scale CRISPR-Cas9 screening in stem cells: theories, applications and challenges

**DOI:** 10.1186/s13287-024-03831-z

**Published:** 2024-07-19

**Authors:** Heng Zhou, Peng Ye, Wei Xiong, Xingxiang Duan, Shuili Jing, Yan He, Zhi Zeng, Yen Wei, Qingsong Ye

**Affiliations:** 1 Center of Regenerative Medicine and Department of Stomatology, Renmin Hospital of Wuhan University, Wuhan, 430060 People’s Republic of China; 2 Institute of Regenerative and Translational Medicine, Tianyou Hospital of Wuhan University of Science and Technology, Wuhan, 430064 Hubei People’s Republic of China; 3 Department of Oral and Maxillofacial Surgery, Massachusetts General Hospital, Harvard Medical School, Boston, MA 02114 USA; 4 The Key Laboratory of Bioorganic Phosphorus Chemistry and Chemical Biology (Ministry of Education), Department of Chemistry, Tsinghua University, Beijing, 100084 People’s Republic of China; 5 Department of Pathology, Renmin Hospital of Wuhan University, Wuhan, 430060 People’s Republic of China; 6 Department of Pharmacy, Renmin Hospital of Wuhan University, Wuhan, 430060 People’s Republic of China

**Keywords:** CRISPR Cas9 screen, Stem cells, CRISPRi, Stem cell therapy, Organoid

## Abstract

Due to the rapid development of stem cell technology, there have been tremendous advances in molecular biological and pathological research, cell therapy as well as organoid technologies over the past decades. Advances in genome editing technology, particularly the discovery of clustered regularly interspaced short palindromic repeats (CRISPR) and CRISPR-related protein 9 (Cas9), have further facilitated the rapid development of stem cell researches. The CRISPR-Cas9 technology now goes beyond creating single gene editing to enable the inhibition or activation of endogenous gene loci by fusing inhibitory (CRISPRi) or activating (CRISPRa) domains with deactivated Cas9 proteins (dCas9). These tools have been utilized in genome-scale CRISPRi/a screen to recognize hereditary modifiers that are synergistic or opposing to malady mutations in an orderly and fair manner, thereby identifying illness mechanisms and discovering novel restorative targets to accelerate medicinal discovery investigation. However, the application of this technique is still relatively rare in stem cell research. There are numerous specialized challenges in applying large-scale useful genomics approaches to differentiated stem cell populations. Here, we present the first comprehensive review on CRISPR-based functional genomics screening in the field of stem cells, as well as practical considerations implemented in a range of scenarios, and exploration of the insights of CRISPR-based screen into cell fates, disease mechanisms and cell treatments in stem cell models. This review will broadly benefit scientists, engineers and medical practitioners in the areas of stem cell research.

## Introduction

Over the past decade, with the discovery of induced pluripotent stem cells (iPSCs), the research on stem cells has been rapidly developed [1]. There is a burgeoning interest that specific genes cause phenotypic alterations, such as guiding directed differentiation, organoids, apoptosis or other programmed modes of death [2, 3]. Next generation sequencing (NGS) has emerged as a vital tool in stem cell research, offering insights into genomic expression changes under various selective pressures or conditions [4–6]. However, functional studies that elucidate the link between these genetic variations and disease pathogenesis have not kept pace with the rapid technological advancements.

Gene knockout or inhibition has been an important way to study the function of certain genes. The advent of RNA interference (RNAi) technology has simplified the process of targeting RNA for translational repression, enabling the study of gene functions through lentiviral delivery or transient transfection [7]. However, it is inefficient to knock out a single gene of interest to study its function, which is subsequently applied in several organisms worldwide to generate RNAi libraries and silence most genes in the genome, so as to achieve genome-wide functional loss screening [8]. While the potential and application of genome-wide RNAi screening has attracted many researchers. The RNAi technology has some intrinsic technical drawbacks, including the inherent incompleteness of protein depletion by RNAi, confounding off-target effects and risk of reporting false positives [9, 10]. Hence, researchers are beginning to explore novel manners that permit direct modification of genome instead of manipulating gene expression, such as zinc finger nucleases (ZFNs) and naturally occurring transcription activater-like effector nucleases (TALENs) [11, 12].

The clustered regularly interspaced short palindromic repeats (CRISPR) and CRISPR-related Protein 9 (Cas9) techniques are among the most flexible and powerful genome editing tools found in the immune system of bacterial [13]. Cas9 is a capable RNA-directed DNA-restricted endonuclease that produces double strand breaks (DSBs) of DNA for gene function studies and therefore plays an critical role within the study of driving mutations and the development of targeted therapies and immunotherapies [14]. Genome-wide CRISPR-based screen present new strategy that has been widely used in cancer and other diseases [15–18]. It is achieved by arranging or merging single guide RNA (sgRNA) libraries with Cas nucleases, which enables high-throughput gene knockout for targeted gene function exploration [19, 20].

This review summarizes the characteristics, tool selection, application scenarios, and strategies of CRISPR-based screen techniques in stem cell research. In addition, potential applications, future challenges, and limitations of this technology in stem cells are presented. Specific usage methods and bioinformatics analysis methods can be referred to other reports.

## CRISPR Cas9 screening and tool selection

**Fig. 1:**
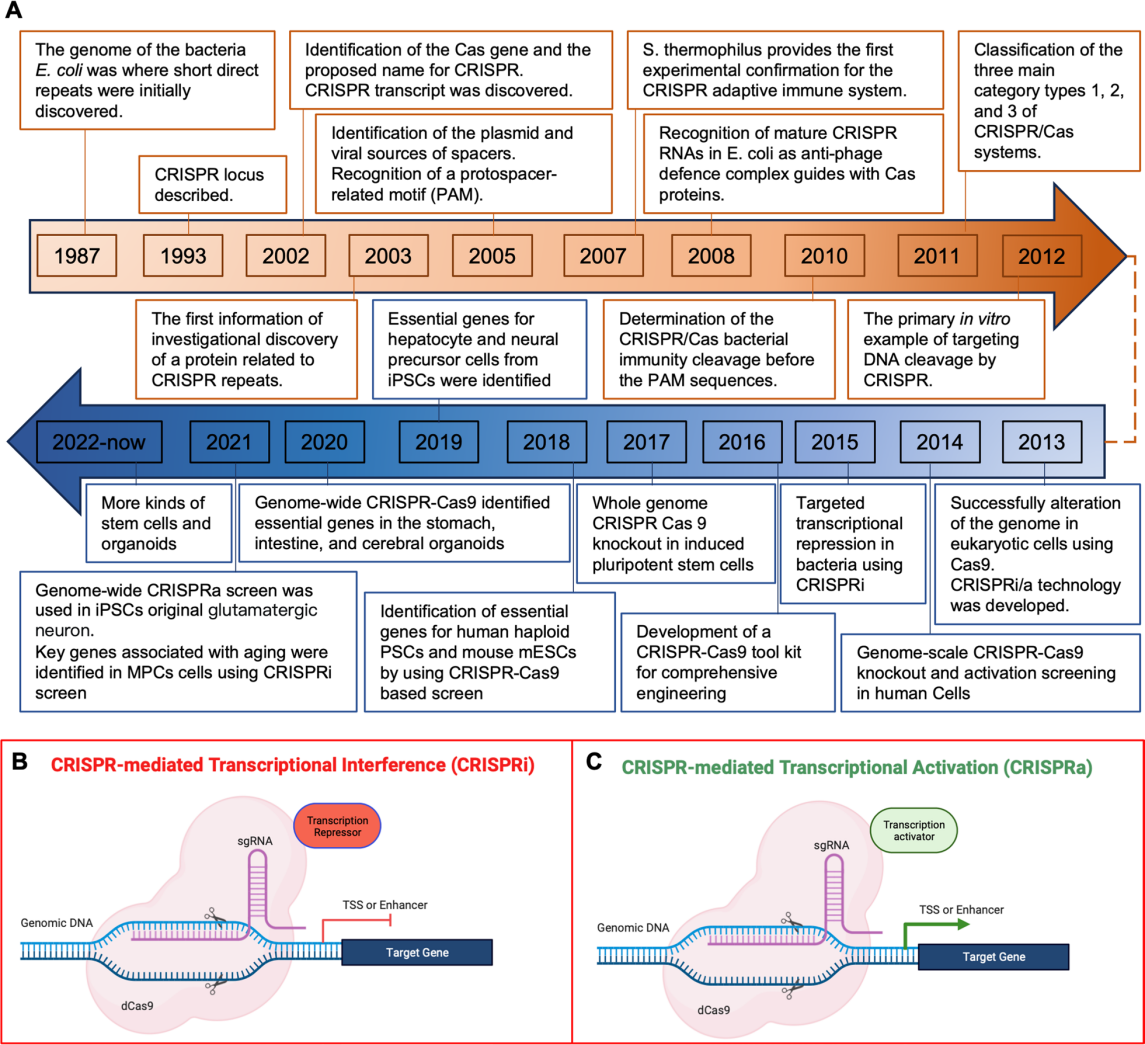
CRISPRi and CRISPRa dependent gene regulation. **A** The progression of the CRISPR/Cas screen systems in stem cell researches and applications over time. **B** CRISPR interference or inhibition (CRISPRi). Under the guidance of gRNA, dCas9-transcription factor fusion protein binds to the transcription start site (TSS) or enhancer of the target gene to inhibit transcription initiation and silence the expression of the target gene. **C** CRISPR activation (CRISPRa). dCas9 binds to different transcriptional activators to up-regulate the expression of target genes. In order to better improve the level of overexpression, multiple transcriptional activators can be recruited to target gene TSS sites or enhancers to improve the activation efficiency

The CRISPR technology is an anti-viral defense system based on the bacterial immune system, characterized by DNA elements called CRISPRs and Cas proteins. CRISPR produces RNA that acts with the bacterial Cas protein to cut the phage genome after bacterial cell infection. This system was later used to cut specific sequences in the mammalian genome, and it revolutionized biomedical research and the development of gene therapy [21, 22]. Scientists also developed multiple CRISPR-based platforms have been developed for the detection of viral sequences [23, 24]. The CRISPR-Cas9 system consists of two biological components: chimeric sgRNA and RNA-guided DNA endonuclease Cas9 [13]. Cas9 loaded with sgRNA is directed to the 20 bp region of the DNA target by base pairing. The target DNA should be immediately preceded with the 5 'NGG sequence (N is any nucleotide) for functional gene editing, called the proto-spacer adjacent motif (PAM) [14, 25]. Cas9 facilitates genome editing by redirecting to target regions and inducing DSBs at target genomic sites. Then cellular mechanisms repair DNA DSBs via Non-homologous end joining (NHEJ) or homology-directed repair (HDR) pathways [26]. Variants of CRISPR-Cas9-based technologies include gene editing and controlling gene expression, with CRISPR interference (CRISPRi) and CRISPR activation (CRISPRa) technologies closely related to CRISPR Screening (Fig. 1A).

### CRISPR interference

The transcription suppressor domains (most commonly the KRAB domain, epigenetic modifiers, or Krüppel-associated box,) can be recruited by deactivated Cas9 protein (dCas9) onto transcription start sites of the human genome to inhibit associated gene transcription [27]. This method is called CRISPR jamming, or CRISPRi. CRISPRi is capable of achieving rigorous knockout levels for both coding and non-coding RNA in human cells. It offers a versatile means to study the functional implications of gene expression modulation, including the examination of partially reduced gene expression levels (Fig. 1B) [28]. Based on flexible ability to control gene expression of CRISPRi, it is possible to study the function of basic genes that cannot be knocked out without slaughtering cells, to partially pharmacologically inhibit cell function, or to simulate decreased gene expression at disease states. CRISPRi can selectively knock out specific transcripts of genes with multiple transcription start sites, since it operates at the transcription start site level [29]. Nevertheless, the use of multiple sgRNAs must be approached with caution, as it increases the potential for off-target effects. This risk necessitates careful sgRNA design and thorough validation to ensure specificity and minimize unintended consequences in gene regulation studies.

The problem of off-target effects is widespread in applications of CRISPRi. Depending on the design of constructed sgRNA, the increased number of off-target binding sites promote the probability of dCas9 off-target effect (Table 1) [30]. In organisms with genomes larger than bacteria, sequence-specific off-target effects are more prominent [31, 32]. However, the off-target effects of CRISPRi are much lower than the RNA interference of the previous generation of knockout technologies [33, 34]. The selection of some bioinformatics tools such as E-CRISP [35] and CRISPOR [36] can simplify the selection of target sequences to minimize the occurrence of mismatch or off-target effects.

**Table 1:** Functional genomics screening techniques comparison

Functional Genomics Screening Technique	Toxicity	Off-Target Effect	Gain/Loss-of- Function Type	Reversibility	sgRNA Target Region	Adaptability
RNAi	Relatively low	Present, especially with poor siRNA design	Knock-down	Reversible	Targeting mRNA through small RNA molecules	High
CRISPRi	Low	Present, generally lower than CRISPRn	Knock-down	Reversible	Targeting specific gene regions through gRNA	High
CRISPRn	Potentially high	Potentially high, requires careful gRNA design	Knock-out	Typically irreversible	Targeting regions determined by gRNA design	High
Base editor	Low	Present, generally lower than CRISPRn	Base change	Irreversible	Targeting specific gene sites through gRNA	Moderate
cDNA overexpression	Can cause cellular stress	Low, mainly affecting the expression level of the target protein	Overexpression	Reversible	Not applicable	High
CRISPRa	Low	Present, generally lower than CRISPRn	Activation	Reversible	Targeting promoter regions of specific genes through gRNA	High
ZFNs	Potentially high	Present, requires careful design of zinc-finger proteins	Knock-out	Irreversible	Targeting DNA through specific DNA-binding domains	Moderate
TALENs	Potentially high	Present, can be reduced through careful design	Knock-out	Irreversible	Targeting DNA through specific DNA-binding domains	Moderate

### CRISPR activation

In a groundbreaking development, synthetic transcriptional activator complexes have been engineered in E. coli, seamlessly integrating activated domains with the programmable DNA-binding capabilities of the CRISPR-Cas9 system. This innovation has given rise to the creation of highly specific custom transcription factors (TFs), a technique known as CRISPRa [37]. CRISPRa is capable of driving gene expression of interest at levels appropriate for metabolic engineering to assess functional consequences by recruiting different transcriptional activation domains to target sites of interest [38, 39]. The activation is achieved by employing modified guide RNAs that bring the dCas9 protein—now functioning as an RNA-binding protein—into play. These sgRNAs are designed to fuse with the RNA-binding protein and its activation domain or to integrate directly with dCas9 (Fig. 1C) [39, 40]. In mammalian cell CRISPRa activation programs usually use the second approach, dCas9 is fused into an activation subdomain, most commonly the tetramer of VP16, VP64, which recruits the transcriptionist to the genomic site where dCas9/gRNA binds [41, 42]. Several additional activation enhancers are used to compensate for the relatively low gene activation capacity of dCas9-VP64, such as SunTag, SAM and VPR [43–45]. Existing CRISPRa tools are specific positioned to rapidly execute combinatorial programs of multigene expression targeting engineered promoters and to control optimal expression conditions for objective production [46]. Unlike overexpression based on cDNA transduction, CRISPRa mainly promotes the expression of target genes by activating the promoter region of target genes, making it more suitable for a wide range of gene overexpression screening (Table 1).

Different from CRISPRi, off-target effects of CRISPRa are not usually considered a problem with Cas9 activators [44, 45]. Given the results of previous RNA-seq experiments, and the likelihood that Cas9 will have an off-target site on the promoter of another gene, thus driving abnormal transcription, is very low, that is, off-target effects are not much of a problem [47]. In order to obtain reduced miss efficiency, it can put the promoter of a gene into a gRNA finder, such as WU-CRISPR [48] or sgRNA scorer 2.0 [49] and selecting the gRNA closest to the transcription start site (TSS) [37, 50]. It is recommended that the guide be positioned in the upstream area of the TSS less than 200 bp for best results, but the area greater than 400 bp can also be better results.

### The whole workflow of CRISPR screening

The flexibility of CRISPRi/a-based manipulation of gene expression and the efficiency of CRISPR in generating gene mutations allow for high-throughput CRISPR gene screening [51]. According to the way of screening, CRISPR screening is divided into partial interest pooled gene screening and genome-wide screening. Because a single mutation can be easily identified using the NGS of the corresponding gRNA region, depending on the gene set of interest, the corresponding gene can be silenced or activated, such as related to kinases [52], ubiquitination [53] or neural differentiation [54]. Customizing a small set of genes of interest is possible. Although genome-wide CRISPR screening is commonly performed, it's worth noting that it can be both time-consuming and costly. Among mammals, human and mouse are the most common choices for genome screening. This selection influences not only the construction of the plasmid library but also the bioinformatics analysis methods used to process the data.

In general, for non-customized CRISPR screening, researchers can purchase the required plasmid libraries at Addgene for a small amount of money, either for partial or whole-genome library screening. The coverage of these libraries varies from a few hundred to 200,000 gRNAs and from 3 to 20 gRNAs per gene to maximize the chances of identifying screening hits. The library also includes non-target guidelines, which are used as internal negative controls to evaluate noise in the screen and normalize read counts [55].

**Fig. 2:**
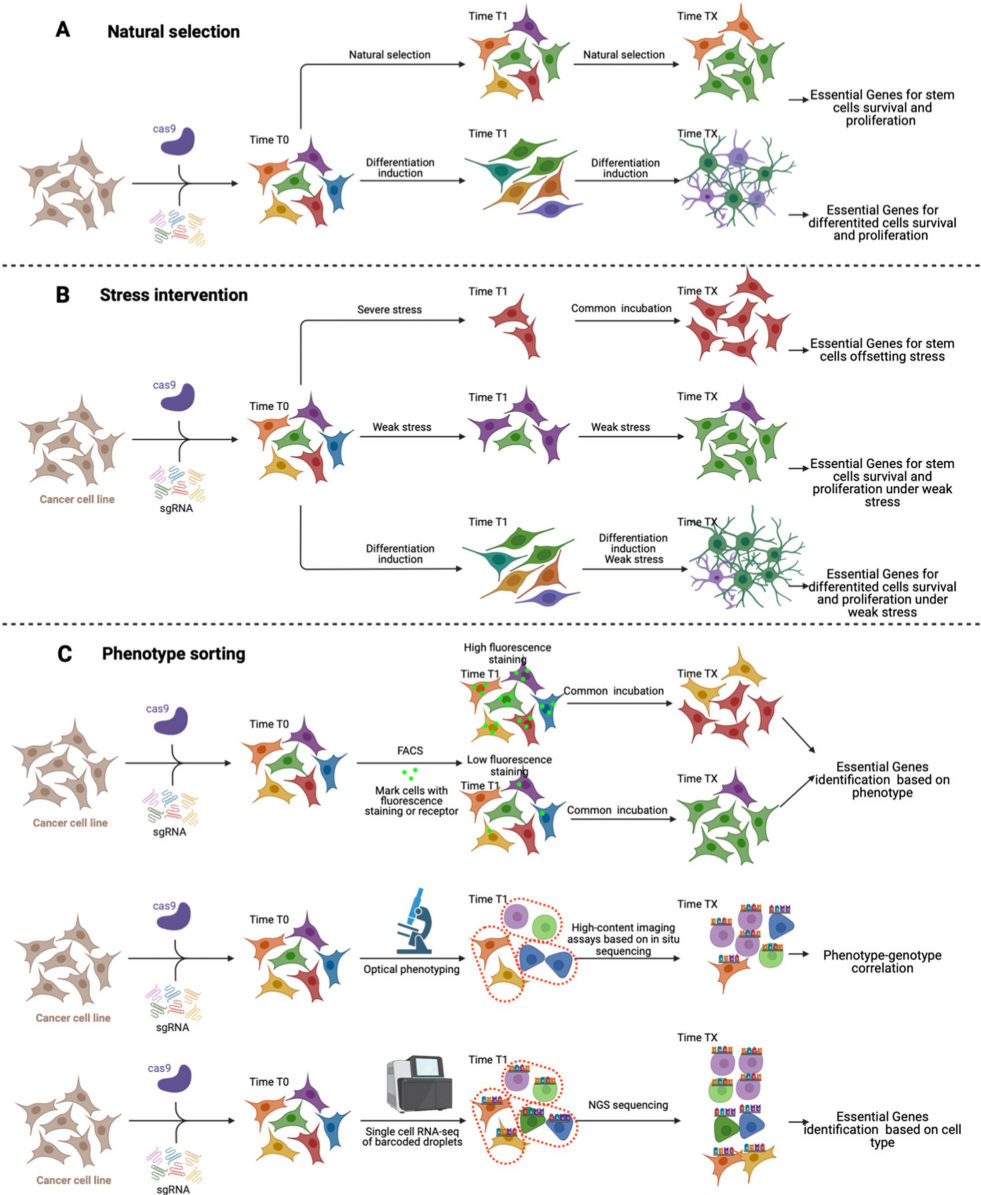
CRISPRi/a screening strategy. After the introduction of CRISPRi/a libraries, purinomycin was used to obtain stem cells with MOI(0.3–0.4) in order to ensure that only a unique lentivirus was infected within each cell. **A** Natural screening. Without the introduction of pressure, the stem cells are obtained by natural selection or induction schemes to induce cell survival/proliferation and then selective pressure is applied to produce key genes that enrich or deplete specific GRnas. **B** Pressure intervention. Appropriate pressure is introduced according to the purpose of the study. This stress can be intense and intense, or it can be prolonged and weak (adaptive screening). Can also be induced under conditions if additional pressure. **C** Phenotypic selection. Based on the cell phenotype of gene expression, pool fluorescence-activated cell sorting (FACS)-based screening protocols were designed, or single-cell RNA sequencing (RNA-seq) and screening were combined to determine the effect of gene perturbations on the high-dimensional transcriptome phenotype. In addition, an ensemble optical screening scheme for genes that distinguish cell phenotypes by high-resolution optical microscopy

Taking genome-wide CRISPR screening as an example, the overall workflow is depicted in Fig. 2. Initially, the entire plasmid library must be amplified. Electroporation is typically employed for this step, as it enhances the efficiency of bacterial transformation compared to the heat shock method [56, 57]. The standard CRISPR library utilizes a single-vector system for lentiviral cell transduction, where each plasmid encodes both the sgRNA and the Streptococcus pyogenes Cas9 (SpCas9). This library design allows for the transduction of any cell type of interest without the need for additional genetic manipulation.

For certain cells that need to be CRISPR screened in vivo or screened for cells that cannot be transduced by lentiviruses, alternative delivery methods such as adeno-associated viruses (AAVs) or retroviruses can be utilized [58, 59]. In instances where long-term differentiation studies are necessary, a degron-based strategy can be employed to modulate Cas9 activity, which may be undesirable during the screening process. This approach provides a flexible means to regulate the duration and intensity of Cas9 expression, thereby facilitating more precise control over the screening procedure [60, 61].

In the case of lentiviral vectors, researchers need to use a low multiple infection (MOI) ratio of 0.3–0.6 for transduction, which represents the number of cells infected with the virus. For more accurate results, an MOI coefficient of less than 0.4 is usually used to ensure as few multiple infections as possible [56]. The uninfected cells are then removed by loading them with an overdose of antibiotics, thereby ensuring that only the successfully modified cells remain. It's crucial to meticulously control the concentration and timing of the antibiotic treatment. Additional doses or treatments may kill some of the already infected cells while avoiding changes in the mutant population unrelated to the phenotype of interest.

After sufficient T0 generation cells were collected for analysis, the investigator was allowed to use positive or negative screening conditions for subsequent study purposes. A negative screening condition usually means leaving the cell in its natural growth state to study which gene knockouts/activations would naturally manipulate the proliferative state of cells. Large libraries of cell lines have been established for researchers to study the effects of genes of interest on cell proliferation or survival. Such as Depmap (https://depmap.org/portal/), PICKLES (https://pickles.hart-lab.org), etc. [62, 63]. A positive screening condition refers to the study of the genetic response to an intervention or insult using conditions that have a significant effect on cell proliferation, such as drugs or genetic manipulation. Such studies are usually done to figure out the important of certain genes for resistance to insult. For positive screening of inhibition of cell proliferation, the differentiated cells are sometimes isolated by flow cytometry or other methods, which will also provide some specific research content, such as organoid studies.

It usually takes three to eight days to collect enough cells once they reach a certain number and pass on the rest. At the end of the experiment, the collected genomic DNA (gDNA) is purified. Using commercial genome isolation kits can greatly speed up the workflow and make them more consistent. Sequencing libraries were generated by amplification of gRNA regions using PCR. PCR primers usually contain a link sequence of NGS and a label that can be assigned to each gDNA sample, so that different amplified samples can be combined for unified sequencing. It is important to note that saturation is prevented by controlling the number of cycles. Some unnecessary amplification will favor more easily amplified gRNA sequences, skew the distribution, and thus lead to a large number of false positive readings. Following NGS and typical QCS, the original read count can be normalized to a total number to account for bias during library expansion.

The results of the NGS yields the initial gRNA counts for each sample, providing a foundation for assessing gRNA enrichment or depletion. Strategies to pinpoint significant hits from CRISPR screens are influenced by several factors, including the library size, the selection protocol employed, and the desired sensitivity of the screen [64]. Since all libraries contain some redundancy, there are usually 4–10 gRNAs for each gene, helping to circumvent potential off-target activity of gRNAs and enhancing the stability of individual gene expression [31, 65, 66]. For subsequent bioinformatics analysis processes, such as MAGeCK (model-based gene essentiality analysis) [67–69], RIGER (RNAi gene enrichment sequencing) [70], BAGEL (Bayes-based gene essentiality analysis) [71], and CARpools (CRISPR analyzers for collection screening) [72] are available. These analytical methods are adept at identifying hits that exhibit significant changes in representation—be it amplified or depleted—across the vast datasets generated by CRISPR screens. Moreover, they facilitate enrichment analyses of gene sets, enabling researchers to adopt a holistic approach to understanding how specific genes influence phenotypes [73, 74]. By leveraging these sophisticated tools, the functional genomics landscape can be navigated with greater precision, revealing the genetic underpinnings of biological processes and disease mechanisms.

### Library tools for genome-scale CRISPR-Cas9 screening

Genome-wide screening libraries leveraging CRISPRi or CRISPRa technologies have evolved through numerous iterations. In the pioneering stages of pooled screening, a substantial number of single-guide RNAs (sgRNAs) were necessary for each gene to counteract the unpredictable variations in gRNA targeting efficacy. The overarching goal of optimization in these screens has been twofold: to enhance the sensitivity of sgRNAs and to minimize off-target effects, thereby increasing the fidelity of the screen. Concurrently, there has been a drive to decrease the average number of sgRNAs per gene, which helps to reduce the complexity of the cell population and simplifies the analysis by focusing on the most effective and specific sgRNAs. Common genome-wide CRISPRi libraries include GeCKOi/a v2 [44, 75], TKOv3 [76, 77], Brie [31], hCRISPRi/a v2 [78], Sabatini/Lander et al. [79]. Sometimes a lab goes through repeated optimizations to maximize the efficiency of the library. Taking the library of the human CRISPR lentivirus developed by Jason Moffat as an example, the first-generation Toronto KnockOut library (TKOv1) contains 176,500 guides (12 guides/genes) targeting 17,661 protein-coding genes [76]. The researchers found that decreasing the library size to more than four gRNAs per gene usually resulted in a small increase in screening sensitivity. Thus, the third-generation TKOv3 library contains only 70,948 sgRNA-targeted 18,053 coding genes, which is convenient for genome-level screening in cell lines while sensitive enough to minimize false negatives in well-designed screens [77].

**Fig. 3:**
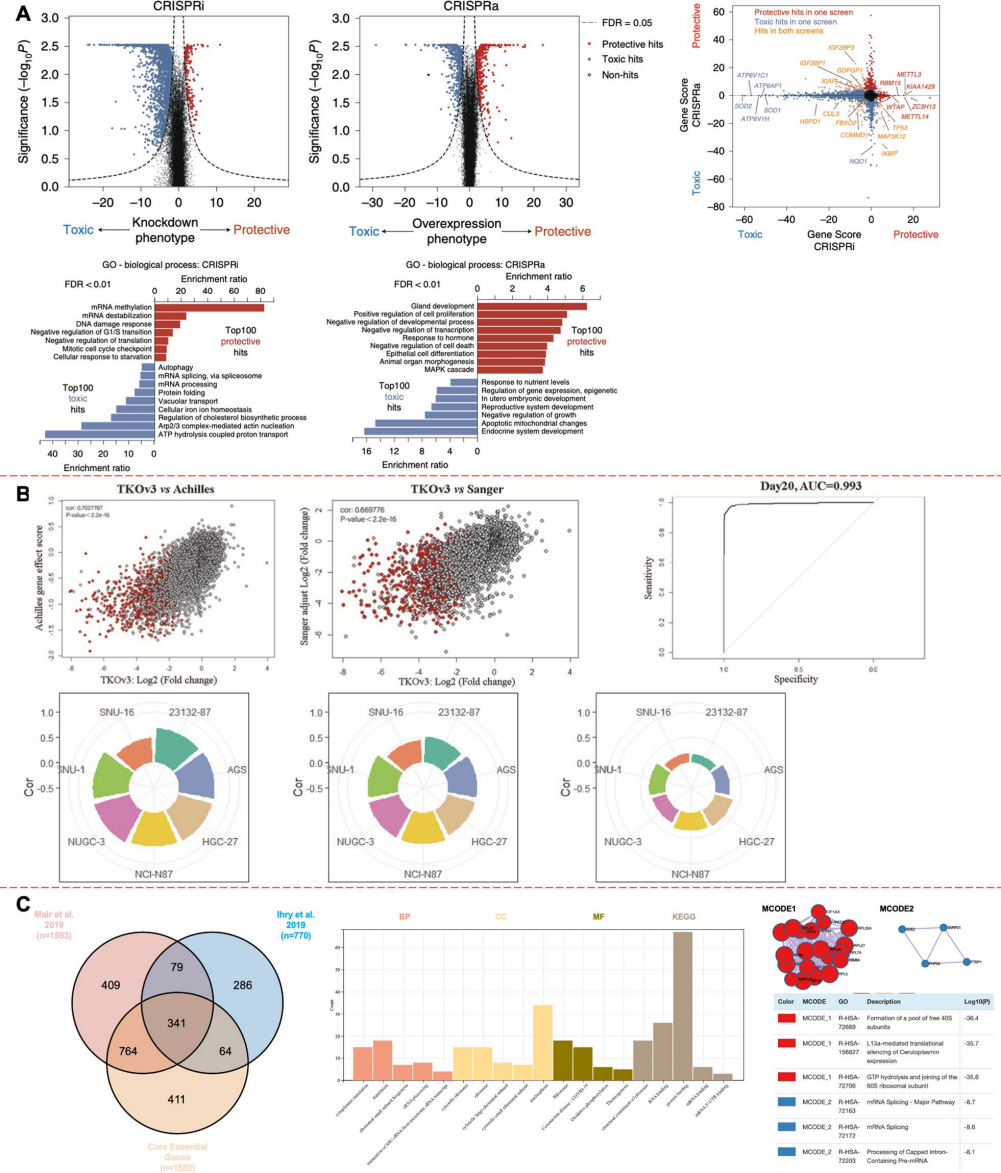
Complementary genetic interpretation for CRISPRi and CRISPRa screen. **A** Knockdown or overexpression phenotypes and statistical significance (two-sided Mann–Whitney U test) for genes targeted in the CRISPRi (left) and CRISPRa (right) screens. Dashed lines show FDR cutoff for hit genes (FDR = 0.05) based on the Gene Score. Comparing Gene Scores for hits from CRISPRi and CRISPRa screens. Hit genes with protective or toxic phenotypes in either screen are shown in red or blue, respectively. Genes that are hits in both screens are shown in orange. GO term enrichment analysis for the top 100 hit genes with protective or toxic phenotypes in CRISPRi (left) and CRISPRa (right) survival screens. Significantly enriched biological process terms (FDR < 0.01) are shown. Copyright from Nature. **B** The comparison of two CRISPR screens datasets in GC cell lines. A Circular barplot of pearson’s correlation coefficient for 16 896 common genes, 1969 common essential genes and 525 common pan-cancer core fitness genes of Achilles and Sanger datasets. [Copyright from Springer Nature]. **C** The Venn diagram shows key Genes in Mair et al. 2019, Ihry et al. 2019 and Core Essential Genes. These 79 overlapping genes were subsequently analyzed for GO and KEGG enrichment. The Protein–protein interaction network and the Molecular Complex Detection were completed

Interestingly, due to the different sensitivities of various pools, they are likely to obtain essential genes (EGs) that do not exactly overlap. For example, Zeng et al., using TKOv3 Library, found that key genes in gastric cancer AGS cell lines did not overlap exactly with Sanger and Achilles datasets, A tRNA N7-methylguanosine (m7G) methyl-transferase (METTL1) is identified as a potential drug target (Fig. 3B) [80]. In particular, for those important genes within the core regions, their overlapping correlations decreased significantly.

### The information convergence of CRISPRi/a screen

It is important to note that the results of CRISPRi screen do not translate directly to a simple reverse interpretation of CRISPRa screen. That is, CRISPRi-screened negative-manipulated EGs do not necessarily produce major overlap with CRISPRa-screened positive controlled EGs. For example, CRISPRi and CRIPSRa screening of human pluripotent stem cells (hPSC) respectively showed great differences in their essential gene sets (Fig. 3A) [81, 82]. Even for some genes, CRISPRa overexpression and CRISPRi knockout will lead to opposite phenotypes, such as desensitization and sensitization to anticancer drugs [83] or bacterial toxins [33]. There are several possible reasons. First, with CRISPRi, not all proteins in the cell are expressed, and some ineffective silencing does not reflect the role of these genes in the cell, while CRISPRa can cause all genes to be overexpressed. Second, the function of some proteins can be compensated by other genes, and even after silencing these genes, the cell can still replicate normally, especially for certain protein complexes. Third, CRISPRa can promote the overexpression of certain cytokines, which has the potential to affect the overall cell culture environment, leading to instability in certain outcomes.

CRISPRi and CRISPRa technologies offer a wealth of information that complements each other in the elucidation of intricate biological pathways. For instance, when applied to the screening of key genes in human myeloid leukemia cells, CRISPRi screening has identified essential housekeeping genes, whereas CRISPRa screening has uncovered a more intricate biological function mapping and the presence of tumor driver genes [33]. In stem cell research, CRISPRa can serve as a substitute for CRISPRi, enabling the study of gene functions that are not typically expressed at specific differentiation times [84]. On the contrary, CRISPRi knockout has greater significance than CRISPRa in the study of the function of individual genes in the protein complex subunit, because overexpression of a gene alone is not necessarily sufficient to cause the phenotype [85]. The nuanced differences between CRISPRi and CRISPRa in their applications highlight the importance of selecting the appropriate tool for the scientific question at hand. Both technologies provide researchers with powerful means to dissect complex biological systems and contribute to a deeper understanding of disease mechanisms and stem cell biology.

## Strategies, methods and schemes of CRISPR-based screen in stem cells

The preceding section delved into the fundamental principles and distinctive features of CRISPR-based screening technology, offering a comprehensive overview of the procedural steps involved. This technology leverages the precision of sgRNA programming to target genes of interest, enabling the exploration of the entire genomic landscape within stem cells across a variety of application contexts. Such screenings can encompass a broad sweep of the genome or zero in on specific classes of functional proteins, thereby elucidating their respective contributions to the intricate functionalities of diverse stem cell types.

Building on this foundation, the subsequent discussion aims to encapsulate and articulate the specific application scenarios of CRISPR screening, presenting detailed examples that highlight the utilization of CRISPR in the context of stem cell research. These examples will illustrate how CRISPR screening is not only a powerful tool for functional genomics but also a versatile platform that can be tailored to address complex biological questions pertinent to stem cell biology and beyond.

### Stem cell research and the role of CRISPR-based screening

The advent of iPSCs has revolutionized stem cell research by providing a means to reprogram adult somatic cells into a pluripotent state, offering new avenues for disease modeling, drug discovery, and personalized medicine [86]. However, the path from understanding stem cell biology to harnessing their therapeutic potential is fraught with challenges, particularly in discerning the molecular cues that guide cell fate decisions and in developing targeted interventions for various diseases.

CRISPR-based screening techniques have emerged as indispensable tools in this quest, offering an unprecedented ability to probe the function of genes at a genome-wide scale. CRISPR (Clustered Regularly Interspaced Short Palindromic Repeats), coupled with the Cas9 nuclease, allows for the precise editing of DNA sequences, enabling researchers to perform both loss-of-function (CRISPRi/Cas9) and gain-of-function (CRISPRa) screens.

These high-throughput screening methods are crucial for several reasons:

Functional Genomics: They enable the functional annotation of the genome by identifying the roles of specific genes in cellular processes, including stem cell differentiation and maintenance.

Cell Fate Determinants: By systematically disrupting or activating genes, researchers can pinpoint determinants of cell fate, which is vital for stem cell differentiation protocols [87].

Disease Mechanisms: CRISPR screens can reveal genes whose mutations contribute to disease pathology, particularly in the context of complex, multifactorial diseases [88].

Therapeutic Target Identification: Understanding the genetic underpinnings of disease through CRISPR screens can lead to the identification of novel therapeutic targets [89].

Drug Response and Resistance: These screens can also illuminate the genetic basis of drug response and resistance, facilitating the development of more effective treatments.

Precision Medicine: By elucidating the genetic landscape that influences stem cell behavior, CRISPR screens contribute to the goal of personalized medicine, where treatments are tailored to an individual's genetic profile.

### Screen without insult

The direct application of CRISPR-based screening in stem cells involves a straightforward strategy: introducing a CRISPRi/a library into the stem cells without subjecting the cells to any additional stress or selective pressures. Following induction, cells are harvested at various time points, and the T0 generation cells are compared with the EGs to conduct the analysis (Fig. 2A). The objective is to identify genes knockout or overexpression can influence the natural growth kinetics of stem cells. This approach mirrors the exploration of tumor cells, where the deletion or overexpression of certain genes can impact the proliferation of various cell types [90, 91]. It is essential to filter out these genes in the final bioinformatic analysis to isolate a unique set of EGs that are specific to the stem cells in question [76, 92].

#### Identification of essential genes in undifferentiated stem cells with natural growth

##### Essential genes in PSCs and ESCs

In the natural screening of stem cells by CRISPR screen technology, multipotential stem cells are the mainstream explored (Fig. 2A). Both pluripotent stem cells (PSCs) and embryonic stem cells (ESCs) are capable to self-renew and be induced to differentiation of most known cell types [93, 94]. Despite the great potentials, utilizing PSCs and ESCs for genetic screening is limited by the expensive and cumbersome cell culture requirements [95] and reduces the efficiency of gene manipulation [81]. However, these cells receive little of the effects of aging on stem cells, they are easier to manipulate when CRISPR libraries are infected, and be also easier to get high-quality data. Tzelepis et al. firstly used CRISPRi to identify all the key genes in mESC. Importantly, they applied an optimized sgRNA vector with higher knock-out efficiency and lower miss efficiency than those using traditional vectors [96]. In 2019, Shohat et al. used another, more frugal, genome-wide CRISPRi library to identify all EGs in mESCs. They also compared their own results with those of Tzelepis et al., and found that their experimental results had a high degree of overlap [97]. This suggests that different sgRNA designs or lentivirus vectors may produce certain bias in the experimental process and experimental results. Therefore, in addition to rational optimization of the CRISPR library, repeated experiments on the same stem cells, as well as additional experimental validations are necessary. In addition, using a small pool of sgRNAs may enable more focused screening of a specific type of gene function. The self-renewal and pluripotency of ESC states are established and maintained by multiple regulatory networks, including TFs and epigenetic regulators (ERs). By aggregating sgRNAs for CRISPR-based screen that target 323 different types of ESC-specific ER and TF genes, two new ERs were identified (TAF5L and TAF6L). TAF5/6L transcription activates Oct4 and c-Myc, as well as their corresponding CORE and MYC regulatory networks, which coordinate gene expression programs to maintain mESC status by controlling self-renewal [98].

Human PSCs (hPSCs) have the ability to generate multiple disease-associated cell types and the potential to enhance preclinical translational efficiency by simulating disease models. In 2019, two research groups simultaneously reported genome-wide CRISPRi screening results for hPSCs. Ihry et al. have identified 770 suitable EGs with a 5% false discovery rate, of which 405 genes overlapped with 1580 core essential genes from cancer cell lines in hPSCs after 18 days of CRISPR-based library exposure [81]. A total of 1593 EGs in hPSCs were specifically identified by Mair et al. [99] used a modified TKOv3 library CRISPRi screened for 12 cultured days. After removing the common core genes, they identified a total of 640 hPSCs specific EGs. To verify the accuracy of key gene identification of hPSCs cells from two different databases, we compared the hPSCs specific sets of the two groups and found that they overlapped a total of 420 EGs. By removing 1580 common genes that influence cell proliferative activity, 79 genes were left that were hPSCs specific to EGs that influence their inventory and proliferation (Fig. 3C). After GO and KEGG enrichment analysis of these genes, we found that ribosome related genes were widely enriched (Fig. 3B). Protein–protein interaction (PPI) network analysis suggested that these genes were highly correlated, and the core genes included RPL5, RPL14, RPL7A, RPL27, RPS16, RPS25, etc. The subsequent Molecular Complex Detection (MCODE) analysis revealed that the 79 genes were divided into two main modules (red and blue modules). The red module mainly concentrated in formation of a pool of free 40S subunits. The blue module focused on mRNA splicing. These results suggest that the functions of hPSCs are closely related to EGs and ribosome. Importantly, our analysis shows that this technique has some reproducibility in stem cell research, but it is not entirely reliable, suggesting that second-hit experiments or further validation of key genes is necessary. Furthermore, Liu et al. developed a CRISPRi library targeting 16,401 lncRNA loci (10 sgRNAs per TSS) and screened for genes required for cell growth and survival in seven human cell types including iPSCs. These large-scale screenings, combined with extensive validation studies, significantly increased the number of lncRNA genes known to have biological functions and revealed that lncRNA functions are highly cell type specific [100].

##### Essential genes in haploid stem cells

Because haploid cells contain only one set of chromosomes, these cells allow for genetic screening by producing highly enriched libraries of hemizygous mutations [101]. Haploid cells showed similar characteristics to PSCs in terms of gene expression, epigenetic profile, alkaline phosphatase activity and colony morphology. Haploid hESCs can be induced to differentiate into haploid somatic cells, producing cell types that represent the three embryonic germ layers. Haploid hPSCs can grow for more than 30 generations under standard culture conditions while maintaining a normal haploid karyotype [102]. Therefore, haploid hPSCs provide an effective CRISPR-based screening platform for solving some unique gene screening problems. Yilmaz et al. used complete CRISPRi screening of haploid hPSC to identify key genes and identify the role of the key P53-mTOR signaling pathway in the survival of haploid hPSC [103]. Bar et al. used CRISPR/Cas9 loss of function screening to identify key genes in parental imprinting involved in haploid and diploid parthenogenetic hESCs, and revealed that ATF7IP is an important suppressor of a group of paternal expression genes [104].

Another specific application is to create a unique diploid genome-wide heterozygous deletion library by fusing a genome-wide mutation library of haploid hESCs with wild-type hESCs. By constructing a genome-wide heterozygous function loss library of hESCs, the aim of discovering genes with haploid dysfunction in these stem cells. These unique cells were able to discover and characterize genes that haploid dysfunction influences hESCs activity and growth, identify dose-sensitive cellular compartments and pathways, and elucidate haploid dysfunction effects in human cells [105]. In a study of totipotent stem cells (TSCs), the removal of TEAD1, a crucial regulator, was found to hinder the cells' proliferation and maintenance, as well as their differentiation into aggressive EVT lineages. Given that TSCs originate from PSCs, a comparison of key genes identified through previous PSC screenings revealed only a 50% overlap. The study also discovered novel regulators in TSCs, including ARID5B, TCAF1, and TEAD1, which had not been previously recognized in this context [106].

#### Identification of essential genes in stem cells under differentiated induction

Stem cells hold a pivotal role due to their remarkable ability to be differentiated into specific cell types both in vitro and in vivo. This characteristic makes them an invaluable tool for investigating cell fate transformations and for developing models for human diseases. PSCs, such as embryonic stem cells (ESCs), have the potential to differentiate into all cell types of the body, while multipotent mesenchymal stem cells (MSCs) are more restricted, typically differentiating into a variety of cell types within the mesodermal lineage, such as bone, cartilage, and fat. Consequently, it is a strategic approach to harness stem cells to explore the fate of EGs under various conditions of directed differentiation [107, 108]. Several studies have delved into the differentiation trajectories of neurons [54, 87, 109, 110], macrophages [111], liver cells [112], cardiac cells [113–115] and more using CRISPR screening technology. In terms of methodology, the prevailing experimental protocols typically involve the introduction of CRISPR screening libraries in undifferentiated PSCs or other stem cells, followed by MOI manipulation, and collecting the cells at T0 generation [116, 117]. The remaining cells were then induced to differentiate into specific mature cells, and the EGs of the differentiated cells were analyzed by sequencing after one or several collection of these cells (T1 to TX) in the differentiation process. (Fig. 2A).

##### Differentiated exploration with IPSCs

Due to the limited proliferation capacity of many maturely differentiated cells, it is difficult to obtain large numbers of such cells by direct culture. The emergence of iPSCs technology allows researchers to obtain a large number of relevant mature cell types. After inducing four TFs (Oct4, Sox2, c-Myc and Klf4) into primary adult cells (such as peripheral blood cells or skin fibroblasts), iPSCs can be generated [118]. The multidirectional differentiation and near-infinite proliferation characters of iPSCs make them ideal for CRISPR-based screen techniques that require large-scale cell culture. IPSCs can be amplified and induced to differentiate into a range of cell subtypes, such as nerve cells, heart muscle cells, liver cells, and even more precise cell subtypes. The tissue analogues and organoids with certain spatial structure formed by three-dimensional (3D) culture in vitro using iPSC, although they are not human organs in the true sense, can simulate real organs in structure and function, can simulate the structure and function of tissues in vivo to the greatest extent, and can be long-term stable subculture [119]. Organoid techniques can better simulate the environment in which cells grow in vivo, thereby revealing more precisely the factors that control selective susceptibility. In uncovering potential therapeutic targets for reducing susceptibility, the use of CRISPR-based screening needs to be implemented in cellular models of relevant diseases. For example, Murakami et al. performed the CRISPR screening using mouse gastric epithelial tissue-like cells, and identify the regulators of WNT-driven stem cell-dependent epithelial renewal in the gastric organoid [120]. In addition, CRISPR-based screening has also been used for EGs screening in the colorectal organoid, and TGF-b-mediated growth restriction [121].

##### Limitation in differentiated genetic exploration

The genetic exploration of the ultimate cell differentiation fate, however, has distinct limitations. Initially, when PSCs undergo directed differentiation, the expression levels of some genes that may be altered cannot be precisely controlled. This is particularly challenging when inducing neural differentiation, as the variety of neuronal subtypes makes it difficult to ensure that stem cells fully differentiate into a specific type of nerve cell in vitro. Organoids exemplify this complexity, often comprising several to many different cell subpopulations in vitro, each with distinct EGs that govern their development, proliferation, and ultimate differentiation fate.

Moreover, maintaining a consistent degree of differentiation during the directed differentiation process is challenging, and the varying sensitivity of cells at different stages to CRISPR screening must be taken into account. Furthermore, individual differences among iPSC donors can affect their differentiation capacity. This suggests that the genetic background of individual donors may influence the gene expression profiles of iPSCs or the post-translational modification of proteins, potentially impacting the accuracy of CRISPR screening [122].

IPSCs are typically derived by reprogramming ordinary cells using abnormal activation of four transcription factors, including the proto-oncogene c-Myc, which endows these cells with the ability for unlimited proliferation and anti-aging capabilities, but this inevitably affects the gene expression profile of the differentiated cells from which they originate [123, 124]. To mitigate the bias caused by such genetic manipulation, some studies should use primary cells for differentiation screening, even though these primary cells are much less proliferative than iPSCs or ESCs [125, 126]. For example, Yu et al. reprogrammed cardiac fibroblasts into cardiac precursor cells in a chemically induced manner and used CRISPRi screening to identify Dmap1 as the key regulatory gene in reprogramming [127].

### CRISPR screening technique with stress intervening

This represents another typical application strategy for CRISPR screening technology, which, in concept, enables screenings predicated on the sensitivity to induced stress conditions. Such a screen design is adept at unearthing EGs that confer sensitivity or resistance to specific insults, a feature with broad applications in medical-related research. Initially, this strategy was primarily employed in cancer studies, facilitating survivor-based screenings conducted under conditions that mimic toxic damage.

This damage can arise from cytotoxic compounds known for their potent cytotoxicity against cancer cells, such as anticancer drugs. Genetic modification screening in this context can elucidate the drugs' mechanisms of action, identify potential biomarkers for patient stratification, and uncover possible avenues of drug resistance. Additionally, the damage can be induced by detrimental environmental factors like hypoxia or exposure to X-rays [128]. Under such conditions, the cytotoxic effect often needs to be greater than 50%, a threshold informed by previous genetic screening studies and indicative of heightened screening sensitivity (Fig. 2B) [129].

Beyond merely discovering novel therapeutic targets that sensitize cells to similar insults, these screenings are particularly instrumental in unraveling the biological mechanisms underlying chemoresistance. They lay the groundwork for the concept of synthetic lethality and the capacity to delineate and quantify the genetic factors contributing to resistance and sensitization.

#### Severe stress intervening for cell survival exploration

The application of insult screening to stem cell research can enrich the research content and obtain more genetic information, because it allows the intervention environment or the survival state of stem cells to mimic the disease, which has important implications for the study of the decisive factors of disease progression, countermeasures and gene modification schemes for stem cell transplantation. Under great stress of insult, the studied stem cells or mature cells die in large numbers. Only a few adversarial genetic manipulations can increase the sensitivity of these cells to the environment. In some cases, the pressure of screening may not be suitable for a long period of time, and after the pressure is removed, the retained cells are expanded to maintain the number required for sequencing (Fig. 2B). Therefore, the purpose of this screening strategy is more to obtain gene modification methods and strategies to resist the harsh survival environment, and their positive screening results are often more critical.

It is important to note that there must be adequate regulation in the implementation of stress, because the results of such implementation will be over-extended. The response of cell lines to stress depends on the genomic condition of the cells, which have many passenger mutations and driver mutations that maintain cell growth. Resistance/sensitization screening usually uses a fixed injury dose, which is predetermined by cell viability tests or the establishment of a dose–response curve. Even two cell lines with the same driver mutation do not necessarily respond in the same way to drug treatment. In order to normalize gene and chromosome replication events, copy number correction is often required [130]. In addition, consider that cell proliferation is a reading that is easily affected by drug dose, medium changes, density, and culture length. Therefore, the determination of multiple algebras is essential in the screening process [131]. The process employs a tiered ranking system to pinpoint the most critical genes and signaling pathways, which is a strategic approach to distill the most impactful findings from the vast data generated by genome-wide screens. Furthermore, downstream validation is an indispensable component of the process, reinforcing the reliability of the initial screen. This can be effectively achieved through viral-mediated gene knock-out approaches or via the targeted inhibition of suspect pathways or proteins of interest using small molecules. To bolster the accuracy of the screening results, a multi-faceted validation strategy is essential. This includes conducting repeated screenings across multiple analogous cell lines, such as ESCs and PSCs. Such an approach helps to control for variability and ensures that the findings are not cell line-specific anomalies. Additionally, comparing the new results with established datasets serves as a crucial step in validating the findings. This comparative analysis not only enhances confidence in the data analysis but also situates the new findings within the broader context of existing scientific knowledge.

In a classic example, in order to investigate the role of iron overload and ferroptosis in neural cells, Ryan et al. used FeSO_4_ and RSL3, an inhibitor of GPX4, to induce iron death in human immortalized microglia cells [132]. Another example is the screening of hit genes for neuronal tolerance under dipeptide repeat proteins. Hexanu-cleotide repeat amplification of the open reading frame 72 (C9orf72) on chromosome 9 is the most common genetic cause of Frontotemporal dementia and amyotrophic lateral sclerosis. DPR is the main driving factor of C9ORF72-related neuronal toxicity. The IPSC-derived DIV79 neurons were treated with PR20 peptide to simulate DPR toxicity. About 70–80% of the progeny of iCas9-iPSC DIV80 neurons died under PR20 treatment. Hence, the identification of CRISPR Cas9 screen suggested important hits against tolerance under dipeptide repeat proteins. Importantly, downstream experiments demonstrated that knock-down of NIMA-related kinase 6 (NEK6) prevented neuronal toxicity caused by poly(PR) [133].

#### Weak injury stress for cell proliferation and survival

The pathological microenvironment in many diseases is characterized by a backdrop of chronic damage, where the condition is often one of enduring impairment or stress. This can involve the expression of disease-associated genes that impact cell survival or the production of toxic substances by the disease. Such conditions are instrumental in uncovering the cellular pathways that govern stress sensitivity or resistance, thereby shedding light on toxic mechanisms, genetic modifiers, host factors of viruses, or even potential therapeutic targets. Harnessing the multipotent capabilities of stem cells, researchers can induce a variety of cell types that mimic the sensitive nature of cells under such pathological conditions. Human mesenchymal progenitor cell (hMPCs) has been used as models of Werner syndrome and Hutchinson-Gilford progeria syndrome to promote cellular aging and performed a CRISPRi-based genome-wide loss of function screening to try to identify novel pro-aging genes. Since a typical feature of cellular senescence is a slowdown in growth rate, the CRISPR-Cas9 screening platform is well suited to identify senescent-associated EGs. Lack of KAT7, a histone acetyltransferase, mitigated cellular aging, and KAT7 was the most popular gene in both presenile hMPC models [134]. Interestingly, they used the CRISPRa screening platform to identify SOX5 overexpression as having an important anti-aging effect at the same in vitro models [135].

They can give the critical genes of insult a better screening environment to get a more accurate gene pool. Obviously, this property of stem cells can promote them to better simulate the development conditions in pathological states, which is an excellent platform for CRISPR Cas9 screening technology (Fig. 2B).

#### Differentiated cells under insult stress intervening

Many genetic diseases manifest as pathological changes that specifically target certain cell types. Consequently, when modeling these diseases, it is essential to obtain the relevant specific cell subsets. This can be effectively achieved through directed differentiation using patient-derived iPSCs or via insult screening with adult stem cells (Fig. 2B). The application of CRISPR Cas9 screening in studying Zika virus (ZIKV) infection has been significant, particularly in identifying key host genes. ZIKV is known to cause neuropathies and severe fetal brain abnormalities when mothers are infected early in pregnancy. Human neural progenitor cells are highly sensitive to ZIKV, leading to viral protein accumulation, virion release, and cell death. Li et al. used CRISPR on iPSC-derived NPs to investigate how ZIKV transmits in nerve cells and found protective effects from knocking out genes related to heparin sulfation, endosomal-lysosome acidification, ER protein complexes, Golgi glycosylation, and negative IFN response regulators [136]. A separate study with glioblastoma stem cells identified gene targets like SSR3, STT3A, MMGT1, SSR2, EMC2, EMC3, and EMC6, some of which were also found in iPSC-derived NP screenings [137].

In addition, functional gene screening for specific differentiated cells under stressful environments is also an important application scheme. CD8 + T cells were extracted from mice, and after entering the CRISPRi screening pool, these T cells were subjected to acute (IL2) and chronic (IL2 + αCD3) stimulation to observe gene regulatory changes in T cell depletion. These screenings also uncovered new genes with a surprising enrichment of epigenetic factors involved in chromatin and nucleosome remodeling, including the cBAF and INO80 complexes, which in many cases limit T cell persistence to a greater extent than previously identified genes [138].

### Cell subpopulation scheme

Stem cells are subjected to additional disturbances such as differentiation, immune response and stress response, and the intracellular gene expression profile is significantly altered, which makes it possible to analyze proliferation potential based on certain gene expression subtypes when screening for proliferation benefits. Most cell subtype functions are attributed to specific changes in protein expression or activity, whether by activation of gene transcription, translation, or post-translational modification processes. Therefore, with appropriate labeling, the ideal cell subpopulation can be obtained and subsequent analysis can be performed. This screening scheme relies on a screening strategy that amplifies or increases the resolution of these changes enough that two or more cell populations can be defined.

#### Screening scheme based on flow cytometry

Phenotypic screening, facilitated by flow cytometry and fluorescence-activated cell sorting (FACS), offers a powerful means to assess the expression of specific cell antigens. Following infection with a CRISPR library, cells can be specifically labeled using antibodies conjugated to fluorescent markers that target both cell surface and intracellular proteins. This approach enables researchers to not only track but also quantify the expression levels of various proteins. More importantly, it allows for the classification of cell populations based on their phenotypic profiles as defined by these protein expressions. By leveraging the precision of flow cytometry and FACS, scientists can dissect complex cellular responses and identify the molecular underpinnings of specific phenotypes with unprecedented clarity and efficiency (Fig. 2C). The most common application of this method in stem cells is the identification of EGs in differentiated subpopulations. Since the induced regimen cannot confirm all differentiation into one cell type, selective bias tends to occur to the final pool gene if further screening is not performed. For example, iPSC-derived kidney organoids, marked by EPCAM, were sorted into epithelial and stromal cells using flow cytometry. CRISPR Cas9 screening highlighted BMP and WNT pathways' roles and a link to Notch signaling [139]. In neural stem cell research, PAX6 + cells were isolated, yielding numerous protein-coding and lncRNA genes. Tracking with fluorescent reporters is beneficial for observing extended cellular processes like differentiation and maturation [109]. Crucially, the capacity to monitor temporal changes with fluorescent reporters offers a distinct advantage over endpoint analysis-based fitness screens. This capability is invaluable for tracking long-term cellular processes such as cellular differentiation or the maturation of immune cells.

#### Single cell sequencing

Chimeric screening protocols that leverage single-cell sequencing and CRISPR-pooled screening offer the capability to identify a greater number of cell subpopulations with single-cell resolution. These methods provide a distinct advantage over FACS and traditional flow cytometry sorting strategies, delivering enhanced flexibility and sensitivity, and enabling the simultaneous identification of multiple groups. For instance, by assessing tens of thousands of individually perturbed single cells that are key to developmental and immune response pathways, the pivotal roles of Cebpb and Irf8 in governing diverse medullary infiltrations have been elucidated [140].

The fusion of single-cell sequencing and CRISPR screening technologies paves the way for conducting extensive functional genomics research at the single-cell level. This powerful combination enables the identification of key genes that are pivotal in governing cell fate and lineage decisions. In the realm of disease research, the synergy of these technologies, when integrated with the diverse differentiation capabilities of stem cells, facilitates the construction of more precise disease models [141]. It offers insights into the cellular heterogeneity underlying diseases and brings to light subpopulations of cells associated with the disease phenotypes. Furthermore, this integrated approach allows for the dissection of complex cellular interactions, shedding light on their roles within intricate biological processes. This includes developmental pathways, the dynamics of immune responses, and other vital physiological mechanisms. This refined strategy not only advances our understanding of cellular heterogeneity but also paves the way for more nuanced and targeted therapeutic interventions in the future of regenerative medicine and immunology.

#### Integrated optical screen.

FACS may struggle to identify low-expressed markers, and the link between molecular profiles, such as RNA, and cell function can often remain elusive. Moreover, traditional single-cell molecular analyses are typically destructive, hindering the continuous monitoring of individual cells' dynamics. The optical pool screening technique circumvents these limitations, employing microscopy to phenotype cells initially and then ascertaining the molecular disruptions for each cell, thus providing a more comprehensive assessment of cellular responses over time [142]. The sgRNA expressed in each cell was then identified by introducing CRISPR-pooled targeted in-situ sequencing. In situ sequencing uses enzymatic amplification to produce high signal levels, allowing imaging at low magnification, which allows millions of cells to be screened from a pooled library. This method greatly expands the range of phenotypes suitable for large-scale pool perturbation screening in mammalian cells.

## Challenges and potential applications of CRISPR-Cas9 screen in stem cell research

The array of genetic exploration techniques including CRISPR-Cas9 screening, Genome-Wide Association Studies (GWAS), RNA-seq, single-cell RNA sequencing (scRNA-seq), and spatial transcriptomics, each brings a unique approach to the study of genetics. The demand for these technologies is expansive, with a notable impact on stem cell research. CRISPR-Cas9 screening technology facilitates in-depth gene function analysis, precise gene editing, and comprehensive genomic structural profiling. Its high-throughput capabilities allow for the rapid identification of links between genetic elements and phenotypic outcomes, a notable advantage over traditional genomic methods that merely report on gene expression variations consequent to phenotypic alterations. To encapsulate, CRISPR-Cas9 screening is adept at revealing a trove of driver genes that are intricately connected to specific research goals.

At present, the technology in this area of research is still very limited. Most of the researchers focused on muti-potent stem cells (PSCs and ESCs) and their differentiation systems, among which Dr. Kampmann's team represented the application of IPSC neural differentiation and explore the key genes that influence pathological changes under stress has given brain science a lot of inspiration. In addition, they have created CRISPRbrain (https://crisprbrain.org), a public resource for brain research based on CRISPR-pooled screen technology, which organizes the genetic screening results of different research groups for different phenotypes in different human cell types. Therefore, promoting the full popularization of this technology in the field of stem cell research is conducive to the overall progress of stem cell research. Based on our current knowledge, we summarize and predict the possible role and potential application model of CRISPR-based screen technology in stem cell research.

### Reveal the essential genes for stem cell maturation and differentiation fate

#### Genetic screening challenges in adult cells

Alvarez-Dominguez and Melton gave a definition of cell maturation, suggesting that metazoan cells develop specialized physiological and morphological characteristics in order to function fully as fate dictates and decides. To the extent that these features represent a fully mature organism or a relatively stable part of its life, this specialized cell is called a “mature cell” [143].

Cell maturation is guided by signals from the environment, which can be divided into chemical (nutrients, oxygen, and growth factors) and physical (mechanical, spatial, and electrical) triggers. Manipulating these triggers in an in vitro culture environment can artificially induce cell differentiation. In fact, with stem cells, as the cells mature, they go from totipotent stem cells to PSCs to monopotent stem cells. This causes their gene expression profile to gradually change. As a result, the genes that affect stem cell survival change significantly. Even for totipotent stem cells, there are not many key genes that overlap. This may be due to the specificity of the cell, but it may also be related to the error of each experiment. Given these factors, it becomes increasingly important to conduct thorough secondary screening and to replicate experiments across different laboratories. Such rigorous scientific practices are crucial for identifying the truly critical genes that influence stem cell behavior and for uncovering any previously overlooked genetic factors that may play a role in cell maturation and differentiation.

CRISPR-Cas9 screening in stem cells has predominantly been applied to pluripotent varieties, such as iPSCs and ESCs. The applicability of this technology to ASCs has been theoretically constrained by their restricted proliferation. Currently, no studies have explicitly harnessed CRISPR-Cas9 for ASCs, a gap that merits attention. Engaging in such research could elucidate the distinct key genes and regulatory pathways of the most primitive ASCs, differentiating them from pluripotent counterparts. Furthermore, extending CRISPR-Cas9 screening to ASCs may enhance our understanding of genetic diseases with origins in these cells.

**Fig. 4:**
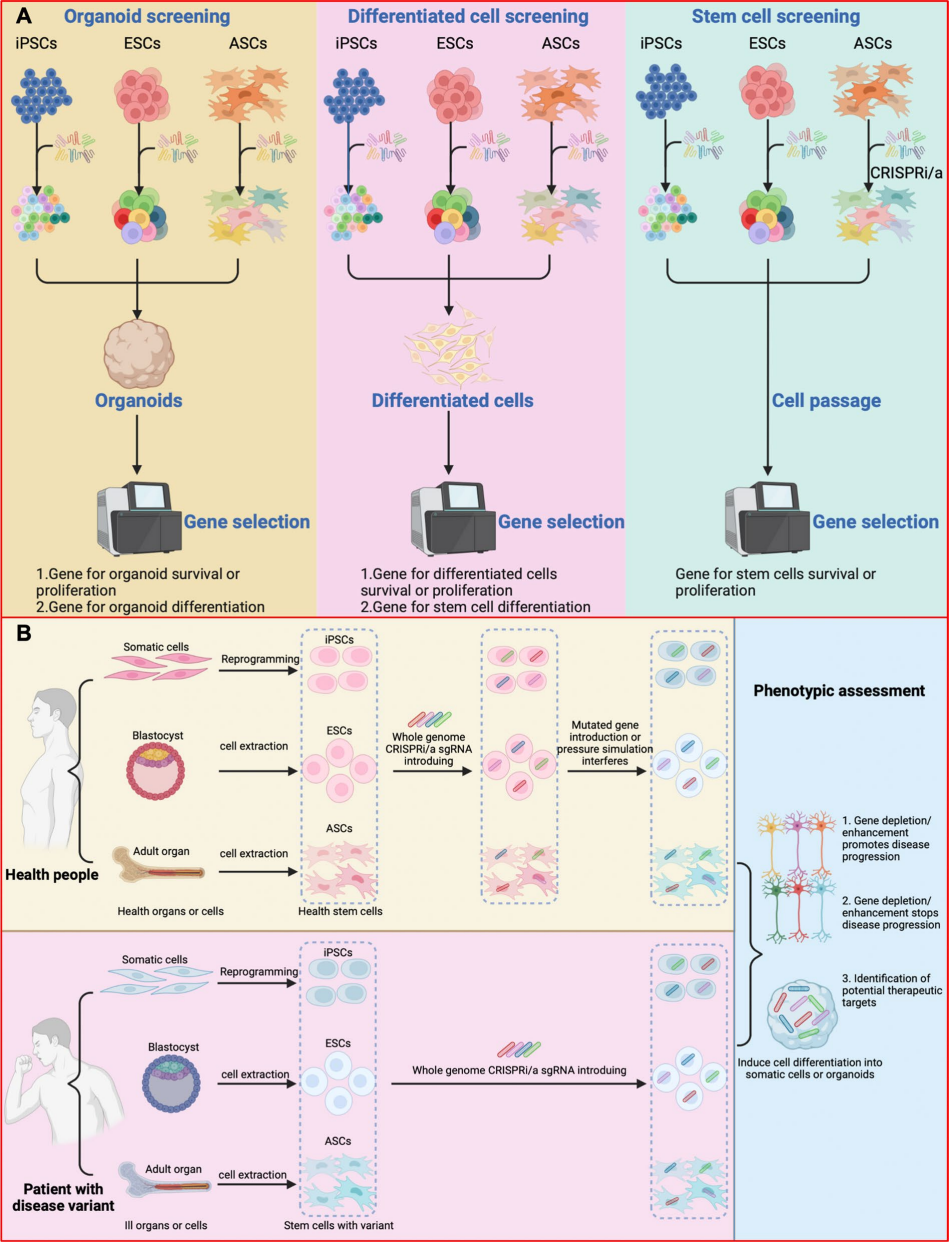
Screen stratedy in stem cells. **A** Induced pluripotent stem cells (iPSCs), embryonic stem cells (ESCs) or adult stem cells (ASCs) are induced with CRISPRi/a library. These stem cells can undergo normal passage to identify essential genes stem cells, or be differentiated into mature cells or organoids under induced conditions. **B** For healthy people, mature and differentiated cells can be extracted from somatic cells and induced pluripotent stem cells (iPSCs) can be obtained through reprogramming. Embryonic stem cells (ESCs) are obtained from blastocyst. ASCs can be obtained from adult organs. After the reintroduction of the CRISPRi/a library, the simulated disease changes were simulated by in vitro stress of the disease environment or by the introduction of exogenous genes. For iPSCs, ESCs, or ACSs obtained from the patient itself, no additional supplemental stress is required because the cells themselves carry adult-specific mutations and disease intervention information. Disease cells acquire phenotypic changes by inducing specific adult cells or organoids. Finally, disease promoting/depleting genes and therapeutic targets are detected

**Fig. 5:**
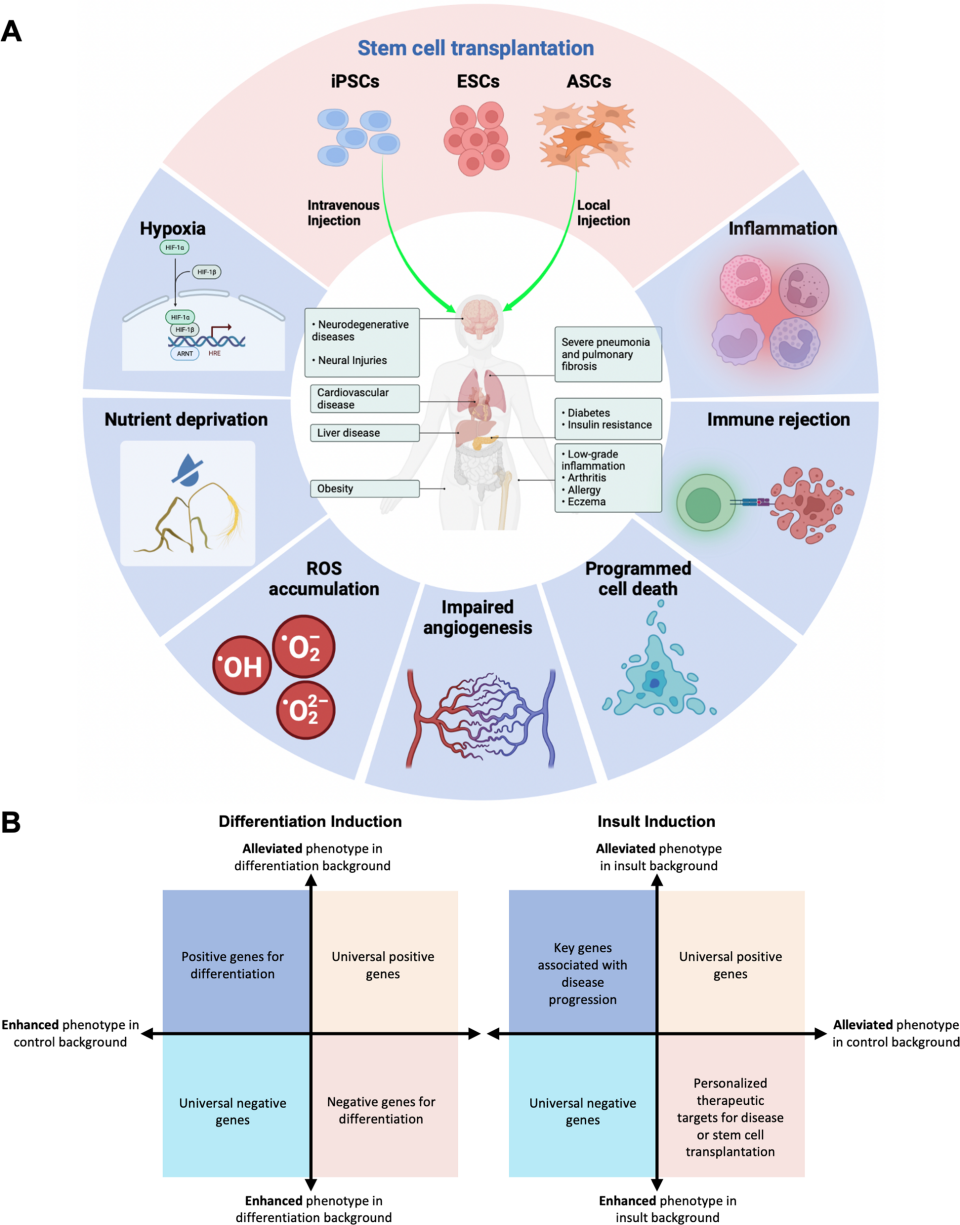
Public threats to the survival of transplanted stem cells. **A** Stem cell therapy is currently proven to be effective in animals and clinical trials for a variety of diseases, including neurodegeneration, nerve damage, heart disease, liver disease, obesity, and more. However, a common problem with stem cell transplants is that the survival rate of stem cells decreases over time. Although different diseases have different etiologies and pathological changes, there are some common problems that threaten stem cell survival, including hypoxia, excessive oxidative stress, inflammation, nutrient deprivation, angiogenesis disorders, programmed cell death, and immune rejection. Using CRISPR screening technology can effectively screen these key genes in the process of stem cell transplantation, so as to find appropriate gene modification programs and treatment strategies. **B** In natural screening, by setting normal passage cells as the control, the induced stem cells can be divided into alleviated phenotypes and enhanced phenotypes. The pooled genes are divided into four gene classes. In general, it is necessary to expel common essential genes (EGs) and select cell-specific EGs as subsequent targets. Modification strategies for specifying disease mechanisms, therapeutic targets, and stem cell transplantation by adding additional insults. It is also necessary to implement screening strategies for more important genes by setting up background controls to identify stronger disease-associated phenotypes observed in the context of the disease. This can lead to personalized therapeutic targets or gene modification strategies suitable for stem cell transplantation, or to the analysis of important gene enrichment that promotes disease progression

The in vitro differentiation of iPSCs, ESCs, or ASCs into specific adult cell types provides critical insights into the molecular mechanisms that govern stem cell differentiation under physiological conditions, as well as the regulatory factors that influence their maturation process [144]. This approach is invaluable for developmental biology studies. For instance, the knockout of certain genes may lead to a swift depletion of the differentiated cell type in question, while leaving other stem cells unaffected (Fig. 4A). The important hits that are screened are defined as specific genes that affect cell development (Fig. 5B). While this type of research is relatively straightforward, a significant consideration is that the extent of stem cell differentiation induced can significantly impact the final analysis. Hence, establishing optimal maturation conditions is essential to ensure that all stem cells are effectively guided to differentiate into the desired target cells [145]. Additionally, employing cell sorting techniques to differentiate between mature and immature cell subtypes offers a strategic alternative. This method allows for a more refined analysis by isolating specific cell populations, thereby enhancing the accuracy and reliability of the study's findings.

#### CRISPR-based screen in organoids

Organoids mimic living organs in both structure and function, which allows researchers to gain a deeper understanding of the development, physiology and pathology of human organs. There are two approaches to building organoids, the first of which involves using tissue stem cells/progenitor cells. Hans Clevers and his team firstly reported that leucine-rich G-protein-coupled receptor-5 (LGR-5) -positive stem cells containing repeats can generate three-dimensional intestinal organoids [146]. Since then, many other organoids, such as the liver, kidneys, pancreas, stomach, uterus, prostate, and breast, have been created from their respective tissues using ASCs [147–150]. The second approach involves the use of PSCs, including ESCs and iPSCs. PSCs can differentiate into three kinds of germ-layer cells through signaling pathways such as WNT, retinoic acid (RA), fibroblast growth factors (FGFs), BMPs and transforming growth factors (TGFs). In recent years, organoids of intestine, liver, lung, thyroid, pancreas, brain, retina, kidney and other organs have been prepared by using PSCs [119].

The advent of organoids has proven to be a more sophisticated approach for studying organ development and creating disease models in vitro than traditional stem cell directed differentiation methods. This is because organoids take into account the intricate interactions between different cell groups, thereby providing a more holistic representation of organ microenvironments. Hence, cell specificity must be considered when conducting organoid screening studies with CRISPR Cas9 screening, which obviously affects the final screening results. Utilizing techniques such as FACS, single-cell, or optic-based screening schemes can help achieve the necessary level of specificity. For example, the basic functional unit of the kidney is nephron, which consists of the glomerulus, a complex of vascular capillaries and podocytes responsible for blood filtration, and a multi-segment tubular epithelium responsible for reabsorption and hormone secretion. Ungricht et al. used EPCAM as a universal marker when conducting CRISPR-based screen for key hits related to kidney organoid development and disease, focusing on stroma and tubular chambers [139]. Although this protocol simplifies the experimental process, the inevitable shortcoming limits blindness to the pattern of podocytes and renal tubules. Other schemes will produce organoids with more complex patterns, but are costly, or require complex multiplexing techniques until they can be applied in a genome-wide manner in organoids [151].

The choice of organoid models for CRISPR-based screening must be deliberate, taking into account the model's ability to accurately represent organ functions and disease states, as well as the developmental processes. The practicality of the experimental design is equally important, considering the large quantity of organoids usually needed. Li et al. exemplified an efficient strategy by employing a focused CRISPR Cas9 pool, targeting only 36 genes, in conjunction with single-cell analysis to explore the neurodevelopmental aspects of depression in brain organoids. This targeted approach minimizes the organoid requirement and, when paired with single-cell methodologies, offers detailed insights into cellular responses to specific gene knockouts and their transcriptional impacts at the single-cell level [152]. In the study of CRISPR-Cas9 screening for intestinal epithelial maturation regulators, the CRISPR-Cas9 used only included 4106 sgRNAs. In addition, double markers (SCA1, CD117) were used to divide intestinal epithelial cells into 3 subgroups for further exploration, which was obviously a great saving amount [121]. However, by selecting some gene groups for study, there will inevitably be gene omission, which is blind to the functional exploration of the whole genome.

### Reveal key genes for disease progression and therapeutic targets

CRISPR-based screening technology is adept at uncovering key mechanisms and fostering the development of innovative treatment strategies for diseases that are linked to an elevated risk due to specific familial inherited conditions or genetic variants. However, the pathways through which these genetic variants contribute to disease pathogenesis are frequently enigmatic or a matter of debate [153]. Gaining a comprehensive understanding of how these disease-associated variations exert their effects is an essential first step in the quest to develop effective therapeutic interventions.

#### Disease-mimicking cells come from patients

Many inherited diseases have a selective susceptibility to specific cell types. For example, autosomal dominant polycystic kidney disease (ADPKD) is the most common inherited kidney disease and is characterized by multiple renal cysts. PKD1 and PKD2 are the main pathogenic genes of ADPKD [154]. However, the expression of ADPKD risk genes is not limited to kidney tissues, suggesting that specific factors in the environment of kidney tissues make them more susceptible to the ADPKD disease process. There are two primary strategies for establishing a phenotype linked to the disease (Fig. 4B). The first strategy involves reprogramming adult patient cells into iPSCs, which carry the natural mutation sites or epigenetic modifications. These diseased iPSCs are then differentiated into the targeted disease-relevant cells. Phenotypic alterations characteristic of the disease would not be observed in other types of control cells, providing a clear distinction. This cell-specific selection of disease phenotypes should closely reflect the phenotypes observed in patients, such as the expression of specific proteins or non-coding RNAs, changes in cell function, cell death, etc. Ideally, this screening should be performed in isogenic cell lines. In the context of ADPKD, cells with PKD1 or PKD2 mutations can be sourced from patient urine cells, peripheral blood mononuclear cells, or skin fibroblasts. These cells can then be reprogrammed into iPSCs [155–157]. The cells harboring allelic mutations are expected to display phenotypic changes once they are induced to differentiate into kidney cells. Subsequently, the disease-associated susceptibility genes or gene clusters that promote the disease can be investigated through the application of CRISPRi/a-based screen techniques.

#### Additional pressure simulates the pathological microenvironment

Another tactic is to add additional insults. For iPSCs from normal sources, external physical, chemical or biological affection make changes in the surrounding environment, which is used to simulate the pathological microenvironment of cells. For example, the brain neurons of Alzheimer's patients face severe amyloid beta deposition or tau entanglement. The use of health iPSCs and the supplement of additional toxic insults, such as Aβs, okadaic acid or even homogenate of cerebrum from transgenic mouse et al. [158] can mimic this pathological change in vitro, thus exploring the molecular mechanism of neuronal change. It is intriguing to note that the suppression of genes directly targeted by a drug or those related to the function of the cell types affected by the drug can enhance the therapeutic efficacy of the drug. This synergistic effect with drug therapy has been a remarkably effective approach in uncovering the mechanisms of drug action. Identifying such drug-gene interactions provides valuable insights into how drugs exert their effects and can guide the development of more targeted treatments. Similarly, genetic modifiers that influence the phenotypes resulting from genetic variations illustrate gene–gene interactions, such as the synthesis of lethal genes in survival screenings. These interactions can shed light on the mechanisms by which genetic variations lead to phenotypic changes. In the context of building models for ADPKD, organoids derived from biallelic mutant iPSCs that have been edited using CRISPR-Cas9 targeting PKD1 are observed to form cyst-like structures within the proximal tubules of the organoids. Furthermore, kidney organoids derived from CRISPR-Cas9 PKD1-edited human iPSC cell lines can be induced to form cysts when stimulated with cyclic adenosine monophosphate (cAMP) [159, 160]. Cyst formation was observed in both renin progenitor cells and collection tube tubules in organoids. Other diseases that are not genetically related also apply to this model, such as Zika virus damage to neurons [136].

#### Genome wide association study combined with CRISPR-based screen

CRISPRi/a technologies offer exquisite control over the expression levels of specific genes in human cells, making them superb tools for deconstructing the intricate gene expression changes that precipitate disease states. Genome-wide association studies (GWAS) frequently identify disease-associated genetic variants located outside of protein-coding regions. Among these are expression quantitative trait loci (eQTLs), a category of genetic loci—predominantly single nucleotide polymorphisms (SNPs)—that can modulate the level of gene expression, consequently influencing the expression of both nearby and distant genes. The impact of eQTLs is contingent upon cell type specificity and the particular state of the cell.

When it comes to a specific disease, even with knowledge of the set of genes whose expression is altered by genetic variation, it is not always straightforward to discern which candidate genes are directly implicated in the disease process [161]. For a particular disease, even when the expression of a set of genes affected by genetic variation is known, it is not always clear which candidate genes are associated with the disease.

Thus, by combining CRISPRi/a screen, it is possible to determine how disease-associated eQTL operate disease processes by influencing specific cells. Mutated cells of the same origin are induced to differentiate into specific tissue cells after being infected with the CRISPRi/a pool, and the phenotypic consequences of individual gene expression changes were evaluated by comparison. Future large-scale screening may study eQTL that affect the expression of many genes, with the goal of revealing convergent phenotypes that point to changes in gene expression most likely to cause disease, as well as the cell types in which they act.

#### Prediction of therapeutic targets

For the introduction of disease-associated insults, it may be more clinically relevant to identify potential therapeutic targets of interest, especially for diseases with unknown genetic factors, which may be a more robust blind screening protocol, such as Amyotrophic Lateral Sclerosis (ALS). In fact, only 10% of people with ALS are genetically related, and there are currently no effective treatment strategies [162]. By introducing CRISPRi/a screening, some unknown drug targets may be discovered. An important motivation for such cell-based disease models is their selection in sequencing phenotypic small molecular sieves for drug discovery. An alternative to pharmacological screening is genetic screening, which aims to identify genetic disorders and improve disease-related phenotypes, thereby pointing to potential therapeutic targets.

Genetic screening presents an enticing alternative for drug screening, capable of illuminating unknown genetic factors that contribute to disease. It offers a direct route to identifying key molecular targets and the mechanisms at play, thereby facilitating the development of potent therapeutic compounds without relying on pre-defined molecular insights. While this method can stand alone, it can also synergize with traditional drug screening, expanding the horizon of potential drug targets. This is particularly evident in oncology, where synthetic lethality research protocols align with this strategy—anticancer agents are crafted to target the weaknesses arising from environmental or genetic perturbations. This dual approach not only enriches drug target discovery but also provides a robust strategy for deciphering the mechanisms behind compounds identified through phenotypic screens [163].

### In vitro environmental model of cell transplantation

#### The survival challenges of stem cell transplantation

Stem cell transplantation is a burgeoning therapeutic avenue for treating conditions such as heart failure, kidney failure, and nerve damage that are unresponsive to traditional clinical treatments. Enhancing the efficacy of these treatments remains a critical area of focus. The challenge of stem cell homing is paramount; whether administered locally or systemically, a significant number of transplanted cells may not reach or persist at the site of injury (Fig. 5A). Furthermore, a harsh microenvironment stemming from poor resident cell survival due to ischemia, hypoxia, oxidative stress, or inflammation is a common denominator across various diseases [164]. In some diseases, specific microenvironmental changes and pathological changes also limit the use of these stem cells. In Alzheimer's patients, for example, large deposits of amyloid beta in the brain not only impair neuronal activity, but can also be toxic to transplanted stem cells [165]. Although advancements in genetic manipulation are beginning to enhance stem cells' adaptability and resilience against such adverse conditions, there is a clear need for more sophisticated and comprehensive screening methods. These protocols would need to address the specific microenvironmental challenges to better understand and improve stem cell survival rates and functionality, thereby unlocking the full potential of stem cell therapies in regenerative medicine [118].

#### Stress strategy for stem cell transplantation environment simulation

Specific experimental setups are ideal for evaluating the resilience of differentiated or transplanted cells facing survival challenges [166], such as those undergoing ferroptosis. For instance, Tian et al. induced a state of chronic oxidative stress in iPSC-derived neurons by removing antioxidants from the culture medium, increasing lipid peroxidation and replicating ferroptosis conditions. Through this, they successfully identified crucial genes related to anti-ferroptosis and lipid peroxidation using comprehensive CRISPRi or CRISPRa screens [167]. Another study studied ferroptosis using IPSC-induced microglia. An iron-dead environment was created by adding iron and RSL3 directly to the medium, and the key hit gene SEC24B was identified using genome-wide CRISPRi technology [132, 168]. In the context of neurodegenerative conditions, iPSC-derived neurons could be exposed to mutations in the tau protein to emulate frontotemporal lobar degeneration. A large-scale screen revealed that reducing CUL5 levels positively modulates tau levels [169], a discovery that contradicts earlier findings in the SH-SY5Y cancer cell line [170]. This suggests that different cells have completely opposite gene function changes in response to the same pathological environment changes. It also shows the importance of repeating multiple cells in the same environment.

For these problems in stem cell transplantation, CRISPRi/a screening undoubtedly has broad application prospects [171, 172]. By introducing CRISPRi/a libraries into stem cells, and then simulating the harsh living environment in vitro, a series of resistance genes can be accurately obtained. As one example, a major challenge in allotransplantation is protecting the graft from immune responses. For stem cell-derived islets (SC-islets) transplantation, Sintov et al. adopted a complementary method to implement scRNA-seq and CRISPR-based genome-wide screening. They found that the JAK/STAT-dependent type II interferon pathway plays an important role in regulating early and late inflammatory response events. Manipulating the activity of the JAK/STAT pathway reduces the immunogenicity of SC-islets, particularly by depleting chemokine ligand 10 (CXCL10) [173]. Other examples are the screening of genes for iron death or chronic liposome dependent oxidative stress mentioned above, or stress from the toxic effects of C9orf72 [133].

Although CRISPR-based genetic screening technology shows excellent promise for gene tampering in stem cell transplants, some concerns must be noted. First, the microenvironment of stem cell transplantation in vivo is more complex, and the reduced survival rate of stem cells is not due to a single factor. However, it is more difficult to simulate the combined effect of multiple factors in vitro. Secondly, stem cell transplantation in vivo will be affected by the microenvironment and gradually tend to differentiate. Different resistance characteristics of different differentiated cells in the process of gene screening will affect the final screening result.

### Ethical limitation associated with CRISPR-based research in stem cells

CRISPR-based stem cell research, particularly involving hPSCs, presents remarkable opportunities for medical breakthroughs. However, it also engenders a multitude of ethical dilemmas. A primary concern is the derivation of hPSCs from embryos, which provokes questions regarding the onset of moral status and personhood of the embryo [174–176].

The application of CRISPR technology for germline editing introduces the potential for heritable genetic modifications within the human genome. This raises significant concerns about the possibility of unintended genetic alterations being inherited by future generations [177, 178]. Safety and efficacy are paramount considerations that must be addressed prior to any clinical application. The safety and efficacy of CRISPR-based interventions require thorough and rigorous testing to mitigate the risk of harm to patients. It is crucial to recognize that the long-term effects of CRISPR-based modifications remain incompletely understood. There is an ethical obligation to undertake long-term studies to fully comprehend their enduring safety and efficacy.

Equity is another critical ethical challenge. Donors must be provided with comprehensive information regarding the procedures, potential risks, and the extensive applications of their donated cells. It is imperative to ensure equitable access to the benefits of stem cell therapies for all individuals, irrespective of their socioeconomic status, to prevent the exacerbation of health disparities. The risk of exploitation, particularly in cases where stem cell donations may originate from vulnerable populations, must be carefully managed.

Addressing these ethical concerns is essential for the responsible progression of CRISPR-based stem cell research. It necessitates a multidisciplinary approach that includes scientists, ethicists, policymakers, and the broader public. This collaborative effort is required to navigate the intricate ethical landscape and ensure that advancements in this field are pursued with the utmost consideration for ethical integrity and societal well-being (Table 2).

**Table 2:** Current studies using CRISPR-based screens in stem cells

Studies	Stem cell type	Differentiated cell type	CRISPR type	Screening strategy	Phenotype	Library size	CRISPR library
2014 Shalem et al. [22]	HUES62	–	CRISPRi	Proliferation	Natural Selection	Whole genome	GeCKO library
2016 Tzelepis et al. [96]	ESC	–	CRISPRi	Proliferation	Natural Selection	Whole genome	v2 library
2017 Liu et al. [100]	iPSCs	–	CRISPRi	Proliferation	Natural Selection	16,401 lncRNA	hCRISPRi-v2.1 library
2018 Yilmaz et al. [101]	hPSCs	–	CRISPRi	Proliferation	haploid	Whole genome	lentiCRISPR v1 library
2018 Ting et al. [92]	HSPC	–	CRISPRi	FACS	Natural Selection	Whole genome	GeCKO v2 library
2018 Li et al. [112]	hiPSCs	Hepatocyte-like cells	CRISPRi	FACS	Hepatocyte differentiation	Whole genome	GeCKO library
2018 Li et al. [116]	mESCs	–	CRISPRi	FACS	Natural Selection	Whole genome	v2 library
2018 Fukuda et al. [117]	mESC	–	CRISPRi	FACS	retroelement silencing	Whole genome	v2 library
2018 Karslioglu et al. [125]	mESC	–	shRNA	FACS	euchromatic state	Whole genome	EXPANDed shRNA library
2019 Yu et al. [127]	Cardiac Fibroblast	Smooth muscle cells	CRISPRi	Proliferation	Cardiac differentiation	Whole genome	v2 library
2019 Li et al. [126]	HUES8	endoderm cells	CRISPRi	FACS	endoderm differentiation	Whole genome	GeCKO/Brunello library
2019 Seruggia et al. [98]	ESCs	–	CRISPRi	FACS	self-renewal	Whole genome	GeCKO library
2019 Shohat et al. [97]	mESC	–	CRISPRi	Proliferation	Natural Selection	Whole genome	v2 library
2019 Ihry et al. [81]	hPSC	–	CRISPRi	Proliferation	Natural Selection	Whole genome	v2 library
2019 Tian et al. [54]	iPSC	Glutamatergic neuron	CRISPRi	Survival	neuronal survival	2000 genes encoding kinases, phosphatases, and drug targets	CRISPRi v2 H1 library
2020 Esk et al. [149]	hESC	Cerebral organoid	CRISPRn	Proliferation	cerebral organoid growth	172 microcephaly candidate genes	private
2019 Mair et al. [99]	hPSC	–	CRISPRi	Proliferation	Natural Selection	whole genome	TKOv3
2019 Li et al. [136]	hiPSC/hESC	Neural progenitor	CRISPRn	Survival	Zika virus	Whole genome	CRISPR Pooled Libraries
2020 Ochiai et al. [144]	mESCs	–	CRISPRi	Proliferation	Natural Selection	Whole genome	GeCKO v2 library
2020 Murakami et al. [120]	corpus and pylorus gastric organoids	–	CRISPRn	Proliferation	gastric organoids culture	Whole genome	GeCKO v2 library
2020 Wang et al. [32]	glioblastoma stem cells	–	CRISPRn	Survival	Zika virus	Whole genome	GeCKO v2 A library
2020 Ringel et al. [150]	intestinal stem cells	intestinal organoids	CRISPRn	Survival	TGF-b activating	Whole genome	LentiCRISPRV2
2020 Lek et al. [153]	MB135-DUX4i	Myoblasts	CRISPRi	Survival	DUX4 activating	Whole genome	GeCKO v2 library
2020 Xu et al. [115]	hESC	Cardiac mesoderm and progenitor	CRISPRn	FACS	Formation of cardiac mesoderm and progenitors	6000 genes	Private
2021 Sapp et al. [113]	hiPSC	Cardiomyocyte	CRISPRn	Survival	Doxorubicin-induced cardiotoxicity	Whole genome	Brunello library
2021 Tian et al. [167]	hiPSC	Glutamatergic neuron	CRISPRi/a	survival	Neuronal survival under normal and oxidative stress conditions	Genome-wide/Ferroptosis	hCRISPRi/a-v2 library
2021 Bar et al. [104]	hpESC	–	CRISPRi	survival	haploid	Whole genome	GeCKO v2 library
2021 Genolet et al. [145]	mESC	–	CRISPRi	FACS	X-chromosomal selection	Whole genome	GeCKOx/xs library
2021 Navarro‐Guerrero et al. [111]	hiPSC	Macrophages	CRISPRi	Survival	macrophage-induction	Whole genome	TKOv3
2022 Ortabozkoyun et al. [166]	mESC	Cervical motor neurons	CRISPRn	FACS	CTCT-boundary-disrupted MNs	Whole genome	GeCKO v2 library
2022 Wu et al. [109]	hiPSC	Neural stem cells	CRISPRi	Proliferation; FACS	cell proliferation during neuronal induction; neural differentiation (PAX6 staining)	Genome-wide libraries against coding and lncRNA genes	CRiNCL sublibraries
2022 Sarel-Gallily et al. [105]	hESC	–	CRISPRi	Survival	haploinsufficient	Whole genome	lentiCRISPR v1 library
2022 Sivakumar et al. [110]	hESC	NPC	CRISPRi	Survival	NPC survival with TP53-/-	Whole genome	GeCKO v2 library
2022 Gao et al. [172]	mESC	N2B27 differentiation	CRISPRi	Proliferation	N2B27 differentiation	Whole genome	GeCKO v2 library
2022 Mamun et al. [173]	mESC	–	CRISPRi	Survival	Stub1 deficiency	Whole genome	GeCKO v2 library
2022 Sintov et al. [170]	hESC	Islet cells	CRISPRi	Survival in vivo	single cell RNA sequence	Whole genome	GeCKO v2 library
2022 Leng et al. [140]	hiPSC	Astrocytes	CRISPRi	FACS/survival	astrocyte differentiation	Whole genome	hCRISPRi-v2
2022 Guo et al. [133]	hiPSC	Cortical neurons	CRISPRn	Survival	PR20 treatment	Whole genome	Brunello library
2022 Dräger et al. [168]	hiPSC	microglia-like cells	CRISPRi	Survival/ proliferation	microglia survival and proliferation	2000 genes encoding kinases, phosphatases, and drug targets	hCRISPRi-v2 H1 library
2022 Cenik et al. [171]	mESC	–	CRISPRi	Survival	SET1A deletion	Whole genome	Brie mouse library
2022 Collier et al. [172]	hPSC	–	CRISPRi	FACS survival/proliferation	nascent naïve PSCs	Whole genome	human v1 CRISPR gRNA library
2022 Dong et al. [106]	hTHSC	–	CRISPRn	survival/proliferation	Natural Selection	Whole genome	Brunello library
2023 Li et al. [152]	miPSC	Cerebral organoid	CRISPRi	sc-seq/survival proliferation	Cerebral organoid-induction	Whole genome	GeCKO v2 library
2023 Adhami et al. [176]	mESC	–	CRISPRi	FACS survival	Germline gene repression	Whole genome	Brie mouse library
2023 Hansen et al. [121]	Proximal small intestine	Mouse intestinal organoid	CRISPRi	FACS/survival proliferation	Intestinal organoid-induction	Whole genome	GeCKO v2 library
2023 Ryan et al. [132]	hiPSC	microglia	CRISPRi	survival/proliferation	Ferroptosis	Whole genome	Guide-it CRISPR Genome-Wide sgRNA Library System
2023 Kaemena et al. [177]	hiPSC	–	CRISPRi	survival/proliferation	reprogramming roadblock	Whole genome	v2 library
2023 Samelson et al. [169]	iPSC	Neurons	CRISPRi	FACS survival/proliferation	Tau V337M heterozygous	Whole genome	hCRISPRi-v2 library
2023 Li et al. [178]	mESC	–	CRISPRi	survival	OGT deletion	Whole genome	Brie mouse library

Abbreviation: miPSC, mouse induced pluripotent stem cells; hiPSC, human induced pluripotent stem cells; mESC, mouse embryonic stem cells; hESCs, human embryonic stem cells; HSPC, hematopoietic stem and progenitor cells; NPC, neural progenitor cells; hTSC, human trophoblast stem cells; FACS, fluorescence activated cell sorting

## Conclusions and future prospects

CRISPR-based genetic screen has wide and significant application strategy in stem cell research, enabling researchers to interrogate almost any biological process and reveal the genetic basis behind it. This offers great potential to identify disease causes and provide potential therapeutic targets. However, many challenges remain.

It is important to note that the screening conditions are very sensitive and can easily distort the final results. As can be seen from our previous summary (Fig. 3), even for the same type of cell, without additional insults or differentiation conditions, the results from different teams have less than half of the hit gene, which may depend on different library sources used, different cell culture environments, or aging interventions. Therefore, we must take the results with the necessary skepticism, so as not to interfere with false positive genes. At the same time, the necessary repetition of experiments from other research groups will further increase the confidence of the results. Another challenge is the limited replication background of ASCs. In general, for genome-wide screening, we need at least 120E6 cells at T0, which makes many ASCs difficult to reach before aging. If adult cells from different individual origin are used, it is likely to interfere with the final screening results by individual differences. In addition, the aging of cells is also the main source of interference. Under the influence of aging, the proliferation of cultured stem cells will be significantly weakened after several generations. Although immortalized cell lines can also reflect the final results of screening to some extent, changes in gene profiles are also likely to affect the screening process. One strategy is to reduce the number of libraries, using custom libraries or libraries with a specific set of functions can effectively reduce the amount of reading. There are also technical challenges. Pooling itself, for example, can have a negative impact on the results of filtering. This is due to the fact that cells cannot grow apart from each other during the screening process, so the cell–cell interaction that compensates for the harmful mutation leads to artificial enrichment of cells carrying that mutation that is not directly related to the original selection criteria. Or some transgenes are gradually silenced during iPSC differentiation, which may prevent CRISPRi/a from continuously perturbing genes.

The summary analysis of the current scope and direction of CRISPR-Cas9 technology suggests that future screening studies will take increasingly sophisticated approaches to better simulate the in-situ growth environment of stem cells or the complex pathological conditions of disease. For example, organoid models are used to simulate the interactions of multiple cells. A more accurate cell reading level is obtained through a more accurate cell sorting scheme. In conclusion, as described in this review, CRISPR-based technologies for extensive screening of combined stem cells have the potential to clarify tissue and organ processes during development and aging, explore important control and therapeutic targets for disease progression, and guide modification strategies for stem cell therapy.

## Footnotes

All statements expressed in this article are solely the author's statements and do not necessarily represent statements from affiliated organizations, nor do they necessarily represent statements from publishers, editors, and reviewers. Any products that may be evaluated in this article, or any claims that their manufacturers may make, are not guaranteed or recognized by the publisher.

## Data Availability

All data are available on request.

## Abbreviations

CRISPR: Clustered regularly interspaced short palindromic repeats
Cas9: CRISPR-related protein 9
dCas9: Deactivated Cas9 proteins
RNAi: RNA interference
ZFNs: Zinc finger nucleases
TALENs: Transcription activater-like effector nucleases
DSBs: Double strand breaks
sgRNA: Single guide RNA
PAM: Proto-spacer adjacent motif
NHEJ: Non-homologous end joining
HDR: Homology-directed repair
CRISPRi: CRISPR interference
CRISPRa: CRISPR activation
TFs: Transcription factors
TSS: Transcription start site
NGS: Next generation sequencing
iPSCs: Induced pluripotent stem cells
SpCas9: S. pyogenes Cas9
MOI: Multiple infection
TKOv1: Toronto KnockOut library
EGs: Essential genes
m7G: N7-methylguanosine
METTL1: Methyl-transferase
hPSC: Human pluripotent stem cells
PSCs: Pluripotent stem cells
ESCs: Embryonic stem cells
ERs: Epigenetic regulators
PPI: Protein–protein interaction
MCODE: Molecular complex detection
C9orf72: Open reading frame 72
NEK6: NIMA-related kinase 6
hMPCs: Human mesenchymal progenitor cell
ZIKV: Zika virus
NPs: Neural progenitor cells
FACS: Fluorescence activated cell sorting
BMP: Bone morphogenetic protein
DCs: Dendritic cells
GWAS: Genome wide association study
ASCs: Adult stem cells
LGR-5: G-protein-coupled receptor-5
RA: Retinoic acid
FGFs: Fibroblast growth factors
TGFs: Transforming growth factors
ADPKD: Autosomal dominant polycystic kidney disease
cAMP: Cyclic adenosine phosphate
eQTL: Expression quantitative trait locus
SNPS: Single nucleotide polymorphisms
ALS: Amyotrophic lateral sclerosis
CXCL10: Chemokine ligand 10

## References

[1] Papapetrou EP. Induced pluripotent stem cells, past and future. Science. 2016;353(6303):991–2. 27701103 10.1126/science.aai7626 PMC5234330

[2] Crespo M, Vilar E, Tsai SY, Chang K, Amin S, Srinivasan T, Zhang T, Pipalia NH, Chen HJ, Witherspoon M, et al. Colonic organoids derived from human induced pluripotent stem cells for modeling colorectal cancer and drug testing. Nat Med. 2017;23(7):878–84. 28628110 10.1038/nm.4355 PMC6055224

[3] Grskovic M, Javaherian A, Strulovici B, Daley GQ. Induced pluripotent stem cells–opportunities for disease modelling and drug discovery. Nat Rev Drug Discov. 2011;10(12):915–29. 22076509 10.1038/nrd3577

[4] Claussnitzer M, Cho JH, Collins R, Cox NJ, Dermitzakis ET, Hurles ME, Kathiresan S, Kenny EE, Lindgren CM, MacArthur DG, et al. A brief history of human disease genetics. Nature. 2020;577(7789):179–89. 31915397 10.1038/s41586-019-1879-7 PMC7405896

[5] Shapiro RM, Kim DDH. Next-generation sequencing-based minimal residual disease monitoring in patients receiving allogeneic hematopoietic stem cell transplantation for acute myeloid leukemia or myelodysplastic syndrome. Curr Opin Hematol. 2018;25(6):425–32. 30281033 10.1097/MOH.0000000000000464

[6] Gallagher MD, Chen-Plotkin AS. The post-GWAS era: from association to function. Am J Hum Genet. 2018;102(5):717–30. 29727686 10.1016/j.ajhg.2018.04.002 PMC5986732

[7] Gutbrod MJ, Martienssen RA. Conserved chromosomal functions of RNA interference. Nat Rev Genet. 2020;21(5):311–31. 32051563 10.1038/s41576-019-0203-6 PMC9478574

[8] Boutros M, Ahringer J. The art and design of genetic screens: RNA interference. Nat Rev Genet. 2008;9(7):554–66. 18521077 10.1038/nrg2364

[9] Jackson AL, Burchard J, Schelter J, Chau BN, Cleary M, Lim L, Linsley PS. Widespread siRNA “off-target” transcript silencing mediated by seed region sequence complementarity. RNA. 2006;12(7):1179–87. 16682560 10.1261/rna.25706 PMC1484447

[10] Echeverri CJ, Beachy PA, Baum B, Boutros M, Buchholz F, Chanda SK, Downward J, Ellenberg J, Fraser AG, Hacohen N, et al. Minimizing the risk of reporting false positives in large-scale RNAi screens. Nat Methods. 2006;3(10):777–9. 16990807 10.1038/nmeth1006-777

[11] Reyon D, Tsai SQ, Khayter C, Foden JA, Sander JD, Joung JK. FLASH assembly of TALENs for high-throughput genome editing. Nat Biotechnol. 2012;30(5):460–5. 22484455 10.1038/nbt.2170 PMC3558947

[12] Miller JC, Tan S, Qiao G, Barlow KA, Wang J, Xia DF, Meng X, Paschon DE, Leung E, Hinkley SJ, et al. A TALE nuclease architecture for efficient genome editing. Nat Biotechnol. 2011;29(2):143–8. 21179091 10.1038/nbt.1755

[13] Hale CR, Zhao P, Olson S, Duff MO, Graveley BR, Wells L, Terns RM, Terns MP. RNA-guided RNA cleavage by a CRISPR RNA-Cas protein complex. Cell. 2009;139(5):945–56. 19945378 10.1016/j.cell.2009.07.040 PMC2951265

[14] Jinek M, Chylinski K, Fonfara I, Hauer M, Doudna JA, Charpentier E. A programmable dual-RNA-guided DNA endonuclease in adaptive bacterial immunity. Science. 2012;337(6096):816–21. 22745249 10.1126/science.1225829 PMC6286148

[15] Wang B, Chen JZ, Luo XQ, Wan GH, Tang YL, Wang QP. The application of genome-wide CRISPR-Cas9 screens to dissect the molecular mechanisms of toxins. Comput Struct Biotechnol J. 2022;20:5076–84. 36187925 10.1016/j.csbj.2022.09.012 PMC9489804

[16] Yi P, Morrow N. Applying CRISPR screen in diabetes research. Diabetes. 2021;70(9):1962–9. 34162685 10.2337/dbi20-0047 PMC9612887

[17] Xue VW, Wong SCC, Cho WCS. Genome-wide CRISPR screens for the identification of therapeutic targets for cancer treatment. Expert Opin Ther Targets. 2020;24(11):1147–58. 32893711 10.1080/14728222.2020.1820986

[18] Yu JSL, Yusa K. Genome-wide CRISPR-Cas9 screening in mammalian cells. Methods. 2019;164–165:29–35. 31034882 10.1016/j.ymeth.2019.04.015

[19] Khaled M, Moustafa AS, El-Khazragy N, Ahmed MI, Abd Elkhalek MA, El Salahy EM. CRISPR/Cas9 mediated knock-out of VPREB1 gene induces a cytotoxic effect in myeloma cells. PLoS ONE. 2021;16(1):e0245349. 33418558 10.1371/journal.pone.0245349 PMC7794028

[20] El-Khazragy N, Ghozy S, Emad P, Mourad M, Razza D, Farouk YK, Mohamed NA, Ahmed MK, Youssef T, Bahnasawy YM, et al. Chimeric antigen receptor T cells immunotherapy: challenges and opportunities in hematological malignancies. Immunotherapy. 2020;12(18):1341–57. 33148070 10.2217/imt-2020-0181

[21] Cong L, Ran FA, Cox D, Lin S, Barretto R, Habib N, Hsu PD, Wu X, Jiang W, Marraffini LA, et al. Multiplex genome engineering using CRISPR/Cas systems. Science. 2013;339(6121):819–23. 23287718 10.1126/science.1231143 PMC3795411

[22] Shalem O, Sanjana NE, Hartenian E, Shi X, Scott DA, Mikkelson T, Heckl D, Ebert BL, Root DE, Doench JG, et al. Genome-scale CRISPR-Cas9 knockout screening in human cells. Science. 2014;343(6166):84–7. 24336571 10.1126/science.1247005 PMC4089965

[23] Jamehdor S, Pajouhanfar S, Saba S, Uzan G, Teimoori A, Naserian S. Principles and applications of CRISPR toolkit in virus manipulation, diagnosis, and virus-host interactions. Cells. 2022;11(6):999. 35326449 10.3390/cells11060999 PMC8946942

[24] Rotondo JC, Martini F, Maritati M, Caselli E, Gallenga CE, Guarino M, De Giorgio R, Mazziotta C, Tramarin ML, Badiale G, et al. Advanced molecular and immunological diagnostic methods to detect SARS-CoV-2 infection. Microorganisms. 2022;10(6):1193. 35744711 10.3390/microorganisms10061193 PMC9231257

[25] McDade JR, Waxmonsky NC, Swanson LE, Fan M. Practical considerations for using pooled lentiviral CRISPR libraries. Curr Protoc Mol Biol. 2016. 10.1002/cpmb.8. 27366891 10.1002/cpmb.8

[26] Sander JD, Joung JK. CRISPR-Cas systems for editing, regulating and targeting genomes. Nat Biotechnol. 2014;32(4):347–55. 24584096 10.1038/nbt.2842 PMC4022601

[27] Thakore PI, D’Ippolito AM, Song L, Safi A, Shivakumar NK, Kabadi AM, Reddy TE, Crawford GE, Gersbach CA. Highly specific epigenome editing by CRISPR-Cas9 repressors for silencing of distal regulatory elements. Nat Methods. 2015;12(12):1143–9. 26501517 10.1038/nmeth.3630 PMC4666778

[28] Schultenkamper K, Brito LF, Wendisch VF. Impact of CRISPR interference on strain development in biotechnology. Biotechnol Appl Biochem. 2020;67(1):7–21. 32064678 10.1002/bab.1901

[29] Kampmann M. CRISPR-based functional genomics for neurological disease. Nat Rev Neurol. 2020;16(9):465–80. 32641861 10.1038/s41582-020-0373-z PMC7484261

[30] Ran FA, Hsu PD, Wright J, Agarwala V, Scott DA, Zhang F. Genome engineering using the CRISPR-Cas9 system. Nat Protoc. 2013;8(11):2281–308. 24157548 10.1038/nprot.2013.143 PMC3969860

[31] Doench JG, Fusi N, Sullender M, Hegde M, Vaimberg EW, Donovan KF, Smith I, Tothova Z, Wilen C, Orchard R, et al. Optimized sgRNA design to maximize activity and minimize off-target effects of CRISPR-Cas9. Nat Biotechnol. 2016;34(2):184–91. 26780180 10.1038/nbt.3437 PMC4744125

[32] Wang G, Sukumar S. Unpredicted central inversion in a sgRNA flanked by inverted repeats. Mol Biol Rep. 2020;47(8):6375–8. 32424520 10.1007/s11033-020-05524-1 PMC13555791

[33] Gilbert LA, Horlbeck MA, Adamson B, Villalta JE, Chen Y, Whitehead EH, Guimaraes C, Panning B, Ploegh HL, Bassik MC, et al. Genome-scale CRISPR-mediated control of gene repression and activation. Cell. 2014;159(3):647–61. 25307932 10.1016/j.cell.2014.09.029 PMC4253859

[34] Gilbert LA, Larson MH, Morsut L, Liu Z, Brar GA, Torres SE, Stern-Ginossar N, Brandman O, Whitehead EH, Doudna JA, et al. CRISPR-mediated modular RNA-guided regulation of transcription in eukaryotes. Cell. 2013;154(2):442–51. 23849981 10.1016/j.cell.2013.06.044 PMC3770145

[35] Heigwer F, Kerr G, Boutros M. E-CRISP: fast CRISPR target site identification. Nat Methods. 2014;11(2):122–3. 24481216 10.1038/nmeth.2812

[36] Concordet JP, Haeussler M. CRISPOR: intuitive guide selection for CRISPR/Cas9 genome editing experiments and screens. Nucl Acids Res. 2018;46(W1):W242–5. 29762716 10.1093/nar/gky354 PMC6030908

[37] Liu Y, Wan X, Wang B. Engineered CRISPRa enables programmable eukaryote-like gene activation in bacteria. Nat Commun. 2019;10(1):3693. 31451697 10.1038/s41467-019-11479-0 PMC6710252

[38] Dong C, Fontana J, Patel A, Carothers JM, Zalatan JG. Synthetic CRISPR-Cas gene activators for transcriptional reprogramming in bacteria. Nat Commun. 2018;9(1):2489. 29950558 10.1038/s41467-018-04901-6 PMC6021436

[39] Bikard D, Jiang W, Samai P, Hochschild A, Zhang F, Marraffini LA. Programmable repression and activation of bacterial gene expression using an engineered CRISPR-Cas system. Nucl Acids Res. 2013;41(15):7429–37. 23761437 10.1093/nar/gkt520 PMC3753641

[40] Zalatan JG, Lee ME, Almeida R, Gilbert LA, Whitehead EH, La Russa M, Tsai JC, Weissman JS, Dueber JE, Qi LS, et al. Engineering complex synthetic transcriptional programs with CRISPR RNA scaffolds. Cell. 2015;160(1–2):339–50. 25533786 10.1016/j.cell.2014.11.052 PMC4297522

[41] Perez-Pinera P, Kocak DD, Vockley CM, Adler AF, Kabadi AM, Polstein LR, Thakore PI, Glass KA, Ousterout DG, Leong KW, et al. RNA-guided gene activation by CRISPR-Cas9-based transcription factors. Nat Methods. 2013;10(10):973–6. 23892895 10.1038/nmeth.2600 PMC3911785

[42] Casas-Mollano JA, Zinselmeier MH, Erickson SE, Smanski MJ. CRISPR-Cas activators for engineering gene expression in higher eukaryotes. CRISPR J. 2020;3(5):350–64. 33095045 10.1089/crispr.2020.0064 PMC7580621

[43] Kampmann M. CRISPRi and CRISPRa screens in mammalian cells for precision biology and medicine. ACS Chem Biol. 2018;13(2):406–16. 29035510 10.1021/acschembio.7b00657 PMC5886776

[44] Konermann S, Brigham MD, Trevino AE, Joung J, Abudayyeh OO, Barcena C, Hsu PD, Habib N, Gootenberg JS, Nishimasu H, et al. Genome-scale transcriptional activation by an engineered CRISPR-Cas9 complex. Nature. 2015;517(7536):583–8. 25494202 10.1038/nature14136 PMC4420636

[45] Chavez A, et al. Highly efficient Cas9-mediated transcriptional programming. Nat Methods. 2015;12(4):326–8. 25730490 10.1038/nmeth.3312 PMC4393883

[46] Fontana J, Sparkman-Yager D, Zalatan JG, Carothers JM. Challenges and opportunities with CRISPR activation in bacteria for data-driven metabolic engineering. Curr Opin Biotechnol. 2020;64:190–8. 32599515 10.1016/j.copbio.2020.04.005

[47] Chavez A, Tuttle M, Pruitt BW, Ewen-Campen B, Chari R, Ter-Ovanesyan D, Haque SJ, Cecchi RJ, Kowal EJK, Buchthal J, et al. Comparison of Cas9 activators in multiple species. Nat Methods. 2016;13(7):563–7. 27214048 10.1038/nmeth.3871 PMC4927356

[48] Wong N, Liu W, Wang X. WU-CRISPR: characteristics of functional guide RNAs for the CRISPR/Cas9 system. Genome Biol. 2015;16:218. 26521937 10.1186/s13059-015-0784-0 PMC4629399

[49] Chari R, Mali P, Moosburner M, Church GM. Unraveling CRISPR-Cas9 genome engineering parameters via a library-on-library approach. Nat Methods. 2015;12(9):823–6. 26167643 10.1038/nmeth.3473 PMC5292764

[50] Fontana J, Dong C, Kiattisewee C, Chavali VP, Tickman BI, Carothers JM, Zalatan JG. Effective CRISPRa-mediated control of gene expression in bacteria must overcome strict target site requirements. Nat Commun. 2020;11(1):1618. 32238808 10.1038/s41467-020-15454-y PMC7113249

[51] Chong ZS, Wright GJ, Sharma S. Investigating cellular recognition using CRISPR/Cas9 genetic screening. Trends Cell Biol. 2020;30(8):619–27. 32595062 10.1016/j.tcb.2020.05.005

[52] Tarumoto Y, et al. LKB1, salt-inducible kinases, and MEF2C are linked dependencies in acute myeloid leukemia. Mol Cell. 2018;69(6):1017-1027.e1016. 29526696 10.1016/j.molcel.2018.02.011 PMC5856641

[53] Jia R, Bonifacino JS. Negative regulation of autophagy by UBA6-BIRC6-mediated ubiquitination of LC3. Elife. 2019. 10.7554/eLife.50034. 31692446 10.7554/eLife.50034 PMC6863627

[54] Tian R, Gachechiladze MA, Ludwig CH, Laurie MT, Hong JY, Nathaniel D, Prabhu AV, Fernandopulle MS, Patel R, Abshari M, et al. CRISPR interference-based platform for multimodal genetic screens in human iPSC-derived neurons. Neuron. 2019;104(2):239-255.e212. 31422865 10.1016/j.neuron.2019.07.014 PMC6813890

[55] Wang T, Wei JJ, Sabatini DM, Lander ES. Genetic screens in human cells using the CRISPR-Cas9 system. Science. 2014;343(6166):80–4. 24336569 10.1126/science.1246981 PMC3972032

[56] Joung J, Konermann S, Gootenberg JS, Abudayyeh OO, Platt RJ, Brigham MD, Sanjana NE, Zhang F. Genome-scale CRISPR-Cas9 knockout and transcriptional activation screening. Nat Protoc. 2017;12(4):828–63. 28333914 10.1038/nprot.2017.016 PMC5526071

[57] Wang T, Lander ES, Sabatini DM. Viral packaging and cell culture for CRISPR-based screens. Cold Spring Harb Protoc. 2016;2016(3):pdb prot090811. 10.1101/pdb.prot090811. 26933250 10.1101/pdb.prot090811 PMC4804706

[58] Henriksson J, Chen X, Gomes T, Ullah U, Meyer KB, Miragaia R, Duddy G, Pramanik J, Yusa K, Lahesmaa R, et al. Genome-wide CRISPR screens in t helper cells reveal pervasive crosstalk between activation and differentiation. Cell. 2019;176(4):882-896.e818. 30639098 10.1016/j.cell.2018.11.044 PMC6370901

[59] Wang G, Chow RD, Ye L, Guzman CD, Dai X, Dong MB, Zhang F, Sharp PA, Platt RJ, Chen S. Mapping a functional cancer genome atlas of tumor suppressors in mouse liver using AAV-CRISPR-mediated direct in vivo screening. Sci Adv. 2018;4(2):eaao5508. 29503867 10.1126/sciadv.aao5508 PMC5829971

[60] Natsume T, Kiyomitsu T, Saga Y, Kanemaki MT. Rapid protein depletion in human cells by auxin-inducible Degron tagging with short homology donors. Cell Rep. 2016;15(1):210–8. 27052166 10.1016/j.celrep.2016.03.001

[61] Senturk S, Shirole NH, Nowak DG, Corbo V, Pal D, Vaughan A, Tuveson DA, Trotman LC, Kinney JB, Sordella R. Rapid and tunable method to temporally control gene editing based on conditional Cas9 stabilization. Nat Commun. 2017;8:14370. 28224990 10.1038/ncomms14370 PMC5322564

[62] Novak LC, Chou J, Colic M, Bristow CA, Hart T. PICKLES v3: the updated database of pooled in vitro CRISPR knockout library essentiality screens. Nucl Acids Res. 2023;51(D1):D1117–21. 36350677 10.1093/nar/gkac982 PMC9825567

[63] Tsherniak A, Vazquez F, Montgomery PG, Weir BA, Kryukov G, Cowley GS, Gill S, Harrington WF, Pantel S, Krill-Burger JM, et al. Defining a cancer dependency map. Cell. 2017;170(3):564-576.e516. 28753430 10.1016/j.cell.2017.06.010 PMC5667678

[64] Bodapati S, Daley TP, Lin X, Zou J, Qi LS. A benchmark of algorithms for the analysis of pooled CRISPR screens. Genome Biol. 2020;21(1):62. 32151271 10.1186/s13059-020-01972-x PMC7063732

[65] Dang Y, Jia G, Choi J, Ma H, Anaya E, Ye C, Shankar P, Wu H. Optimizing sgRNA structure to improve CRISPR-Cas9 knockout efficiency. Genome Biol. 2015;16:280. 26671237 10.1186/s13059-015-0846-3 PMC4699467

[66] Fu Y, Sander JD, Reyon D, Cascio VM, Joung JK. Improving CRISPR-Cas nuclease specificity using truncated guide RNAs. Nat Biotechnol. 2014;32(3):279–84. 24463574 10.1038/nbt.2808 PMC3988262

[67] Wang B, Wang M, Zhang W, Xiao T, Chen CH, Wu A, Wu F, Traugh N, Wang X, Li Z, et al. Integrative analysis of pooled CRISPR genetic screens using MAGeCKFlute. Nat Protoc. 2019;14(3):756–80. 30710114 10.1038/s41596-018-0113-7 PMC6862721

[68] Li W, Koster J, Xu H, Chen CH, Xiao T, Liu JS, Brown M, Liu XS. Quality control, modeling, and visualization of CRISPR screens with MAGeCK-VISPR. Genome Biol. 2015;16:281. 26673418 10.1186/s13059-015-0843-6 PMC4699372

[69] Li W, Xu H, Xiao T, Cong L, Love MI, Zhang F, Irizarry RA, Liu JS, Brown M, Liu XS. MAGeCK enables robust identification of essential genes from genome-scale CRISPR/Cas9 knockout screens. Genome Biol. 2014;15(12):554. 25476604 10.1186/s13059-014-0554-4 PMC4290824

[70] Luo B, Cheung HW, Subramanian A, Sharifnia T, Okamoto M, Yang X, Hinkle G, Boehm JS, Beroukhim R, Weir BA, et al. Highly parallel identification of essential genes in cancer cells. Proc Natl Acad Sci USA. 2008;105(51):20380–5. 19091943 10.1073/pnas.0810485105 PMC2629277

[71] Hart T, Moffat J. BAGEL: a computational framework for identifying essential genes from pooled library screens. BMC Bioinformatics. 2016;17:164. 27083490 10.1186/s12859-016-1015-8 PMC4833918

[72] Winter J, Breinig M, Heigwer F, Brugemann D, Leible S, Pelz O, Zhan T, Boutros M. caRpools: an R package for exploratory data analysis and documentation of pooled CRISPR/Cas9 screens. Bioinformatics. 2016;32(4):632–4. 26508755 10.1093/bioinformatics/btv617

[73] Wang X, Cairns MJ. Gene set enrichment analysis of RNA-Seq data: integrating differential expression and splicing. BMC Bioinform. 2013;14(Suppl 5):S16. 10.1186/1471-2105-14-S5-S16 PMC3622641 23734663

[74] Cantu E, Lederer DJ, Meyer K, Milewski K, Suzuki Y, Shah RJ, Diamond JM, Meyer NJ, Tobias JW, Baldwin DA, et al. Gene set enrichment analysis identifies key innate immune pathways in primary graft dysfunction after lung transplantation. Am J Transplant. 2013;13(7):1898–904. 23710539 10.1111/ajt.12283 PMC3954988

[75] Sanjana NE, Shalem O, Zhang F. Improved vectors and genome-wide libraries for CRISPR screening. Nat Methods. 2014;11(8):783–4. 25075903 10.1038/nmeth.3047 PMC4486245

[76] Hart T, Chandrashekhar M, Aregger M, Steinhart Z, Brown KR, MacLeod G, Mis M, Zimmermann M, Fradet-Turcotte A, Sun S, et al. High-resolution CRISPR screens reveal fitness genes and genotype-specific cancer liabilities. Cell. 2015;163(6):1515–26. 26627737 10.1016/j.cell.2015.11.015

[77] Hart T, Tong AHY, Chan K, Van Leeuwen J, Seetharaman A, Aregger M, Chandrashekhar M, Hustedt N, Seth S, Noonan A, et al. Evaluation and design of genome-wide CRISPR/SpCas9 knockout screens. G3 (Bethesda). 2017;7(8):2719–27. 28655737 10.1534/g3.117.041277 PMC5555476

[78] Horlbeck MA, Gilbert LA, Villalta JE, Adamson B, Pak RA, Chen Y, Fields AP, Park CY, Corn JE, Kampmann M *et al*: Compact and highly active next-generation libraries for CRISPR-mediated gene repression and activation. *Elife* 2016, **5** 10.7554/eLife.19760 PMC5094855 27661255

[79] Park RJ, Wang T, Koundakjian D, Hultquist JF, Lamothe-Molina P, Monel B, Schumann K, Yu H, Krupzcak KM, Garcia-Beltran W, et al. A genome-wide CRISPR screen identifies a restricted set of HIV host dependency factors. Nat Genet. 2017;49(2):193–203. 27992415 10.1038/ng.3741 PMC5511375

[80] Zeng Z, Zhang X, Jiang CQ, Zhang YG, Wu X, Li J, Tang S, Li L, Gu LJ, Xie XY, et al. Identifying novel therapeutic targets in gastric cancer using genome-wide CRISPR-Cas9 screening. Oncogene. 2022;41(14):2069–78. 35177812 10.1038/s41388-022-02177-1

[81] Ihry RJ, Salick MR, Ho DJ, Sondey M, Kommineni S, Paula S, Raymond J, Henry B, Frias E, Wang Q, et al. Genome-scale CRISPR screens identify human pluripotency-specific genes. Cell Rep. 2019;27(2):616–630616. 30970262 10.1016/j.celrep.2019.03.043

[82] Yang J, Rajan SS, Friedrich MJ, Lan G, Zou X, Ponstingl H, Garyfallos DA, Liu P, Bradley A, Metzakopian E. Genome-scale CRISPRa screen identifies novel factors for cellular reprogramming. Stem Cell Rep. 2019;12(4):757–71. 10.1016/j.stemcr.2019.02.010 PMC6450436 30905739

[83] Jost M, Chen Y, Gilbert LA, Horlbeck MA, Krenning L, Menchon G, Rai A, Cho MY, Stern JJ, Prota AE, et al. Combined CRISPRi/a-based chemical genetic screens reveal that rigosertib is a microtubule-destabilizing agent. Mol Cell. 2017;68(1):210-223.e216. 28985505 10.1016/j.molcel.2017.09.012 PMC5640507

[84] Boettcher M, Tian R, Blau JA, Markegard E, Wagner RT, Wu D, Mo X, Biton A, Zaitlen N, Fu H, et al. Dual gene activation and knockout screen reveals directional dependencies in genetic networks. Nat Biotechnol. 2018;36(2):170–8. 29334369 10.1038/nbt.4062 PMC6072461

[85] Ramkumar P, Abarientos AB, Tian R, Seyler M, Leong JT, Chen M, Choudhry P, Hechler T, Shah N, Wong SW, et al. CRISPR-based screens uncover determinants of immunotherapy response in multiple myeloma. Blood Adv. 2020;4(13):2899–911. 32589729 10.1182/bloodadvances.2019001346 PMC7362346

[86] Li K, Ouyang M, Zhan J, Tian R. CRISPR-based functional genomics screening in human-pluripotent-stem-cell-derived cell types. Cell Genom. 2023;3(5):100300. 37228745 10.1016/j.xgen.2023.100300 PMC10203043

[87] Qi S, Sivakumar S, Yu H. CRISPR-Cas9 screen in human embryonic stem cells to identify genes required for neural differentiation. STAR Protoc. 2022;3(4):101682. 36115024 10.1016/j.xpro.2022.101682 PMC9490198

[88] Ahmed M, Muffat J, Li Y. Understanding neural development and diseases using CRISPR screens in human pluripotent stem cell-derived cultures. Front Cell Dev Biol. 2023;11:1158373. 37101616 10.3389/fcell.2023.1158373 PMC10123288

[89] Worthington AK, Forsberg EC. A CRISPR view of hematopoietic stem cells: moving innovative bioengineering into the clinic. Am J Hematol. 2022;97(9):1226–35. 35560111 10.1002/ajh.26588 PMC9378712

[90] Subasri M, Shooshtari P, Watson AJ, Betts DH. Analysis of TERT isoforms across TCGA, GTEx and CCLE datasets. Cancers (Basel). 2021;13(8):1853. 33924498 10.3390/cancers13081853 PMC8070023

[91] Sachdev P, Ronen R, Dutkowski J, Littlefield BA. Systematic analysis of genetic and pathway determinants of eribulin sensitivity across 100 human cancer cell lines from the cancer cell line encyclopedia (CCLE). Cancers (Basel). 2022;14(18):4532. 36139690 10.3390/cancers14184532 PMC9496846

[92] Ting PY, Parker AE, Lee JS, Trussell C, Sharif O, Luna F, Federe G, Barnes SW, Walker JR, Vance J, et al. Guide Swap enables genome-scale pooled CRISPR-Cas9 screening in human primary cells. Nat Methods. 2018;15(11):941–6. 30297964 10.1038/s41592-018-0149-1

[93] Nishikawa S, Jakt LM, Era T. Embryonic stem-cell culture as a tool for developmental cell biology. Nat Rev Mol Cell Biol. 2007;8(6):502–7. 17522593 10.1038/nrm2189

[94] Soldner F, Jaenisch R. Stem cells, genome editing, and the path to translational medicine. Cell. 2018;175(3):615–32. 30340033 10.1016/j.cell.2018.09.010 PMC6461399

[95] Chen G, Gulbranson DR, Hou Z, Bolin JM, Ruotti V, Probasco MD, Smuga-Otto K, Howden SE, Diol NR, Propson NE, et al. Chemically defined conditions for human iPSC derivation and culture. Nat Methods. 2011;8(5):424–9. 21478862 10.1038/nmeth.1593 PMC3084903

[96] Tzelepis K, Koike-Yusa H, De Braekeleer E, Li Y, Metzakopian E, Dovey OM, Mupo A, Grinkevich V, Li M, Mazan M, et al. A CRISPR dropout screen identifies genetic vulnerabilities and therapeutic targets in acute myeloid leukemia. Cell Rep. 2016;17(4):1193–205. 27760321 10.1016/j.celrep.2016.09.079 PMC5081405

[97] Shohat S, Shifman S. Genes essential for embryonic stem cells are associated with neurodevelopmental disorders. Genome Res. 2019;29(11):1910–8. 31649057 10.1101/gr.250019.119 PMC6836742

[98] Seruggia D, Oti M, Tripathi P, Canver MC, LeBlanc L, Di Giammartino DC, Bullen MJ, Nefzger CM, Sun YBY, Farouni R, et al. TAF5L and TAF6L maintain self-renewal of embryonic stem cells via the MYC regulatory network. Mol Cell. 2019;74(6):1148-1163.e1147. 31005419 10.1016/j.molcel.2019.03.025 PMC6671628

[99] Mair B, Tomic J, Masud SN, Tonge P, Weiss A, Usaj M, Tong AHY, Kwan JJ, Brown KR, Titus E, et al. Essential gene profiles for human pluripotent stem cells identify uncharacterized genes and substrate dependencies. Cell Rep. 2019;27(2):599-615.e512. 30970261 10.1016/j.celrep.2019.02.041

[100] Liu SJ, Horlbeck MA, Cho SW, Birk HS, Malatesta M, He D, Attenello FJ, Villalta JE, Cho MY, Chen Y, et al. CRISPRi-based genome-scale identification of functional long noncoding RNA loci in human cells. Science. 2017;355(6320):eaah7111. 10.1126/science.aah7111 PMC5394926 27980086

[101] Yilmaz A, Peretz M, Sagi I, Benvenisty N. Haploid human embryonic stem cells: half the genome Double the Value. Cell Stem Cell. 2016;19(5):569–72. 27814478 10.1016/j.stem.2016.10.009

[102] Sagi I, Chia G, Golan-Lev T, Peretz M, Weissbein U, Sui L, Sauer MV, Yanuka O, Egli D, Benvenisty N. Derivation and differentiation of haploid human embryonic stem cells. Nature. 2016;532(7597):107–11. 26982723 10.1038/nature17408

[103] Yilmaz A, Peretz M, Aharony A, Sagi I, Benvenisty N. Defining essential genes for human pluripotent stem cells by CRISPR-Cas9 screening in haploid cells. Nat Cell Biol. 2018;20(5):610–9. 29662178 10.1038/s41556-018-0088-1

[104] Bar S, Vershkov D, Keshet G, Lezmi E, Meller N, Yilmaz A, Yanuka O, Nissim-Rafinia M, Meshorer E, Eldar-Geva T, et al. Identifying regulators of parental imprinting by CRISPR/Cas9 screening in haploid human embryonic stem cells. Nat Commun. 2021;12(1):6718. 34795250 10.1038/s41467-021-26949-7 PMC8602306

[105] Sarel-Gallily R, Golan-Lev T, Yilmaz A, Sagi I, Benvenisty N. Genome-wide analysis of haploinsufficiency in human embryonic stem cells. Cell Rep. 2022;38(13):110573. 35354027 10.1016/j.celrep.2022.110573

[106] Dong C, Fu S, Karvas RM, Chew B, Fischer LA, Xing X, Harrison JK, Popli P, Kommagani R, Wang T, et al. A genome-wide CRISPR-Cas9 knockout screen identifies essential and growth-restricting genes in human trophoblast stem cells. Nat Commun. 2022;13(1):2548. 35538076 10.1038/s41467-022-30207-9 PMC9090837

[107] Mazziotta C, Badiale G, Cervellera CF, Tognon M, Martini F, Rotondo JC. Regulatory mechanisms of circular RNAs during human mesenchymal stem cell osteogenic differentiation. Theranostics. 2024;14(1):143–58. 38164139 10.7150/thno.89066 PMC10750202

[108] Pittenger MF, Discher DE, Peault BM, Phinney DG, Hare JM, Caplan AI. Mesenchymal stem cell perspective: cell biology to clinical progress. NPJ Regen Med. 2019;4:22. 31815001 10.1038/s41536-019-0083-6 PMC6889290

[109] Wu D, Poddar A, Ninou E, Hwang E, Cole MA, Liu SJ, Horlbeck MA, Chen J, Replogle JM, Carosso GA, et al. Dual genome-wide coding and lncRNA screens in neural induction of induced pluripotent stem cells. Cell Genom. 2022;2(11):100177. 36381608 10.1016/j.xgen.2022.100177 PMC9648144

[110] Sivakumar S, Qi S, Cheng N, Sathe AA, Kanchwala M, Kumar A, Evers BM, Xing C, Yu H. TP53 promotes lineage commitment of human embryonic stem cells through ciliogenesis and sonic hedgehog signaling. Cell Rep. 2022;38(7):110395. 35172133 10.1016/j.celrep.2022.110395 PMC8904926

[111] Navarro-Guerrero E, Tay C, Whalley JP, Cowley SA, Davies B, Knight JC, Ebner D. Genome-wide CRISPR/Cas9-knockout in human induced pluripotent stem cell (iPSC)-derived macrophages. Sci Rep. 2021;11(1):4245. 33608581 10.1038/s41598-021-82137-z PMC7895961

[112] Li S, Li M, Liu X, Yang Y, Wei Y, Chen Y, Qiu Y, Zhou T, Feng Z, Ma D, et al. Genetic and chemical screenings identify HDAC3 as a key regulator in hepatic differentiation of human pluripotent stem cells. Stem Cell Reps. 2018;11(1):22–31. 10.1016/j.stemcr.2018.05.001 PMC6066908 29861165

[113] Sapp V, Aguirre A, Mainkar G, Ding J, Adler E, Liao R, Sharma S, Jain M. Genome-wide CRISPR/Cas9 screening in human iPS derived cardiomyocytes uncovers novel mediators of doxorubicin cardiotoxicity. Sci Rep. 2021;11(1):13866. 34230586 10.1038/s41598-021-92988-1 PMC8260754

[114] Nishiga M, Qi LS, Wu JC. CRISPRi/a Screening with Human iPSCs. Methods Mol Biol. 2021;2320:261–81. 34302664 10.1007/978-1-0716-1484-6_23 PMC11047756

[115] Xu J, Zhou C, Foo KS, Yang R, Xiao Y, Bylund K, Sahara M, Chien KR. Genome-wide CRISPR screen identifies ZIC2 as an essential gene that controls the cell fate of early mesodermal precursors to human heart progenitors. Stem Cells. 2020;38(6):741–55. 32129551 10.1002/stem.3168 PMC7891398

[116] Li M, Yu JSL, Tilgner K, Ong SH, Koike-Yusa H, Yusa K. Genome-wide CRISPR-KO screen uncovers mTORC1-mediated Gsk3 regulation in naive pluripotency maintenance and dissolution. Cell Rep. 2018;24(2):489–502. 29996108 10.1016/j.celrep.2018.06.027 PMC6057492

[117] Fukuda K, Okuda A, Yusa K, Shinkai Y. A CRISPR knockout screen identifies SETDB1-target retroelement silencing factors in embryonic stem cells. Genome Res. 2018;28(6):846–58. 29728365 10.1101/gr.227280.117 PMC5991520

[118] Zhou H, He Y, Xiong W, Jing S, Duan X, Huang Z, Nahal GS, Peng Y, Li M, Zhu Y, et al. MSC based gene delivery methods and strategies improve the therapeutic efficacy of neurological diseases. Bioact Mater. 2023;23:409–37. 36474656 10.1016/j.bioactmat.2022.11.007 PMC9713256

[119] Garreta E, Kamm RD. Chuva de Sousa Lopes SM, Lancaster MA, Weiss R, Trepat X, Hyun I, Montserrat N: Rethinking organoid technology through bioengineering. Nat Mater. 2021;20(2):145–55. 33199860 10.1038/s41563-020-00804-4

[120] Murakami K, Terakado Y, Saito K, Jomen Y, Takeda H, Oshima M, Barker N. A genome-scale CRISPR screen reveals factors regulating Wnt-dependent renewal of mouse gastric epithelial cells. Proc Natl Acad Sci USA. 2021. 10.1073/pnas.2016806118. 33479180 10.1073/pnas.2016806118 PMC7848749

[121] Hansen SL, Larsen HL, Pikkupeura LM, Maciag G, Guiu J, Muller I, Clement DL, Mueller C, Johansen JV, Helin K, et al. An organoid-based CRISPR-Cas9 screen for regulators of intestinal epithelial maturation and cell fate. Sci Adv. 2023;9(28):eadg4055. 37436979 10.1126/sciadv.adg4055 PMC10337909

[122] Kajiwara M, Aoi T, Okita K, Takahashi R, Inoue H, Takayama N, Endo H, Eto K, Toguchida J, Uemoto S, et al. Donor-dependent variations in hepatic differentiation from human-induced pluripotent stem cells. Proc Natl Acad Sci USA. 2012;109(31):12538–43. 22802639 10.1073/pnas.1209979109 PMC3411998

[123] Babiarz JE, Ravon M, Sridhar S, Ravindran P, Swanson B, Bitter H, Weiser T, Chiao E, Certa U, Kolaja KL. Determination of the human cardiomyocyte mRNA and miRNA differentiation network by fine-scale profiling. Stem Cells Dev. 2012;21(11):1956–65. 22050602 10.1089/scd.2011.0357 PMC4048009

[124] Ogawa S, Surapisitchat J, Virtanen C, Ogawa M, Niapour M, Sugamori KS, Wang S, Tamblyn L, Guillemette C, Hoffmann E, et al. Three-dimensional culture and cAMP signaling promote the maturation of human pluripotent stem cell-derived hepatocytes. Development. 2013;140(15):3285–96. 23861064 10.1242/dev.090266 PMC4074277

[125] Bulut-Karslioglu A, Macrae TA, Oses-Prieto JA, Covarrubias S, Percharde M, Ku G, Diaz A, McManus MT, Burlingame AL, Ramalho-Santos M. The transcriptionally permissive chromatin state of embryonic stem cells is acutely tuned to translational output. Cell Stem Cell. 2018;22(3):369-383.e368. 29499153 10.1016/j.stem.2018.02.004 PMC5836508

[126] Li QV, Dixon G, Verma N, Rosen BP, Gordillo M, Luo R, Xu C, Wang Q, Soh CL, Yang D, et al. Genome-scale screens identify JNK-JUN signaling as a barrier for pluripotency exit and endoderm differentiation. Nat Genet. 2019;51(6):999–1010. 31110351 10.1038/s41588-019-0408-9 PMC6545159

[127] Yu JSL, Palano G, Lim C, Moggio A, Drowley L, Plowright AT, Bohlooly YM, Rosen BS, Hansson EM, Wang QD, et al. CRISPR-knockout screen identifies Dmap1 as a regulator of chemically induced reprogramming and differentiation of cardiac progenitors. Stem Cells. 2019;37(7):958–72. 30932271 10.1002/stem.3012 PMC6767549

[128] Hayman TJ, Baro M, MacNeil T, Phoomak C, Aung TN, Cui W, Leach K, Iyer R, Challa S, Sandoval-Schaefer T, et al. STING enhances cell death through regulation of reactive oxygen species and DNA damage. Nat Commun. 2021;12(1):2327. 33875663 10.1038/s41467-021-22572-8 PMC8055995

[129] Bassik MC, Kampmann M, Lebbink RJ, Wang S, Hein MY, Poser I, Weibezahn J, Horlbeck MA, Chen S, Mann M, et al. A systematic mammalian genetic interaction map reveals pathways underlying ricin susceptibility. Cell. 2013;152(4):909–22. 23394947 10.1016/j.cell.2013.01.030 PMC3652613

[130] Meyers RM, Bryan JG, McFarland JM, Weir BA, Sizemore AE, Xu H, Dharia NV, Montgomery PG, Cowley GS, Pantel S, et al. Computational correction of copy number effect improves specificity of CRISPR-Cas9 essentiality screens in cancer cells. Nat Genet. 2017;49(12):1779–84. 29083409 10.1038/ng.3984 PMC5709193

[131] Iorio F, Behan FM, Goncalves E, Bhosle SG, Chen E, Shepherd R, Beaver C, Ansari R, Pooley R, Wilkinson P, et al. Unsupervised correction of gene-independent cell responses to CRISPR-Cas9 targeting. BMC Genomics. 2018;19(1):604. 30103702 10.1186/s12864-018-4989-y PMC6088408

[132] Ryan SK, Zelic M, Han Y, Teeple E, Chen L, Sadeghi M, Shankara S, Guo L, Li C, Pontarelli F, et al. Microglia ferroptosis is regulated by SEC24B and contributes to neurodegeneration. Nat Neurosci. 2023;26(1):12–26. 36536241 10.1038/s41593-022-01221-3 PMC9829540

[133] Guo W, Wang H, Kumar Tharkeshwar A, Couthouis J, Braems E, Masrori P, Van Schoor E, Fan Y, Ahuja K, Moisse M, et al. CRISPR/Cas9 screen in human iPSC-derived cortical neurons identifies NEK6 as a novel disease modifier of C9orf72 poly(PR) toxicity. Alzheimers Dement. 2023;19(4):1245–59. 35993441 10.1002/alz.12760 PMC9943798

[134] Wang W, Zheng Y, Sun S, Li W, Song M, Ji Q, Wu Z, Liu Z, Fan Y, Liu F, et al. A genome-wide CRISPR-based screen identifies KAT7 as a driver of cellular senescence. Sci Transl Med. 2021;13(575):eabd2655. 33408182 10.1126/scitranslmed.abd2655

[135] Jing Y, Jiang X, Ji Q, Wu Z, Wang W, Liu Z, Guillen-Garcia P, Esteban CR, Reddy P, Horvath S, et al. Genome-wide CRISPR activation screening in senescent cells reveals SOX5 as a driver and therapeutic target of rejuvenation. Cell Stem Cell. 2023;30(11):1452-1471.e1410. 37832549 10.1016/j.stem.2023.09.007

[136] Li Y, Muffat J, Omer Javed A, Keys HR, Lungjangwa T, Bosch I, Khan M, Virgilio MC, Gehrke L, Sabatini DM, et al. Genome-wide CRISPR screen for Zika virus resistance in human neural cells. Proc Natl Acad Sci USA. 2019;116(19):9527–32. 31019072 10.1073/pnas.1900867116 PMC6510995

[137] Wang S, Zhang Q, Tiwari SK, Lichinchi G, Yau EH, Hui H, Li W, Furnari F, Rana TM. Integrin alphavbeta5 Internalizes Zika Virus during neural stem cells infection and provides a promising target for antiviral therapy. Cell Rep. 2020;30(4):969-983.e964. 31956073 10.1016/j.celrep.2019.11.020 PMC7293422

[138] Belk JA, Yao W, Ly N, Freitas KA, Chen YT, Shi Q, Valencia AM, Shifrut E, Kale N, Yost KE, et al. Genome-wide CRISPR screens of T cell exhaustion identify chromatin remodeling factors that limit T cell persistence. Cancer Cell. 2022;40(7):768-786.e767. 35750052 10.1016/j.ccell.2022.06.001 PMC9949532

[139] Ungricht R, Guibbal L, Lasbennes MC, Orsini V, Beibel M, Waldt A, Cuttat R, Carbone W, Basler A, Roma G, et al. Genome-wide screening in human kidney organoids identifies developmental and disease-related aspects of nephrogenesis. Cell Stem Cell. 2022;29(1):160-175.e167. 34847364 10.1016/j.stem.2021.11.001

[140] Leng K, Rose IVL, Kim H, Xia W, Romero-Fernandez W, Rooney B, Koontz M, Li E, Ao Y, Wang S, et al. CRISPRi screens in human iPSC-derived astrocytes elucidate regulators of distinct inflammatory reactive states. Nat Neurosci. 2022;25(11):1528–42. 36303069 10.1038/s41593-022-01180-9 PMC9633461

[141] Genga RMJ, Kernfeld EM, Parsi KM, Parsons TJ, Ziller MJ, Maehr R. Single-cell RNA-sequencing-based CRISPRi screening resolves molecular drivers of early human endoderm development. Cell Rep. 2019;27(3):708-718.e710. 30995470 10.1016/j.celrep.2019.03.076 PMC6525305

[142] Feldman D, Singh A, Schmid-Burgk JL, Carlson RJ, Mezger A, Garrity AJ, Zhang F, Blainey PC. Optical pooled screens in human cells. Cell. 2019;179(3):787-799.e717. 31626775 10.1016/j.cell.2019.09.016 PMC6886477

[143] Alvarez-Dominguez JR, Melton DA. Cell maturation: Hallmarks, triggers, and manipulation. Cell. 2022;185(2):235–49. 34995481 10.1016/j.cell.2021.12.012 PMC8792364

[144] Ochiai H, Hayashi T, Umeda M, Yoshimura M, Harada A, Shimizu Y, Nakano K, Saitoh N, Liu Z, Yamamoto T, et al. Genome-wide kinetic properties of transcriptional bursting in mouse embryonic stem cells. Sci Adv. 2020;6(25):eaaz6699. 32596448 10.1126/sciadv.aaz6699 PMC7299619

[145] Genolet O, Monaco AA, Dunkel I, Boettcher M, Schulz EG. Identification of X-chromosomal genes that drive sex differences in embryonic stem cells through a hierarchical CRISPR screening approach. Genome Biol. 2021;22(1):110. 33863351 10.1186/s13059-021-02321-2 PMC8051100

[146] Sato T, Vries RG, Snippert HJ, van de Wetering M, Barker N, Stange DE, van Es JH, Abo A, Kujala P, Peters PJ, et al. Single Lgr5 stem cells build crypt-villus structures in vitro without a mesenchymal niche. Nature. 2009;459(7244):262–5. 19329995 10.1038/nature07935

[147] Clevers H. Modeling development and disease with organoids. Cell. 2016;165(7):1586–97. 27315476 10.1016/j.cell.2016.05.082

[148] Xia Y, Izpisua Belmonte JC. Design approaches for generating organ constructs. Cell Stem Cell. 2019;24(6):877–94. 31173717 10.1016/j.stem.2019.05.016

[149] Esk C, Lindenhofer D, Haendeler S, Wester RA, Pflug F, Schroeder B, Bagley JA, Elling U, Zuber J, von Haeseler A, et al. A human tissue screen identifies a regulator of ER secretion as a brain-size determinant. Science. 2020;370(6519):935–41. 33122427 10.1126/science.abb5390

[150] Ringel T, Frey N, Ringnalda F, Janjuha S, Cherkaoui S, Butz S, Srivatsa S, Pirkl M, Russo G, Villiger L, et al. Genome-scale CRISPR screening in human intestinal organoids identifies drivers of TGF-beta resistance. Cell Stem Cell. 2020;26(3):431-440.e438. 32142663 10.1016/j.stem.2020.02.007

[151] Datlinger P, Rendeiro AF, Boenke T, Senekowitsch M, Krausgruber T, Barreca D, Bock C. Ultra-high-throughput single-cell RNA sequencing and perturbation screening with combinatorial fluidic indexing. Nat Methods. 2021;18(6):635–42. 34059827 10.1038/s41592-021-01153-z PMC7612019

[152] Li C, Fleck JS, Martins-Costa C, Burkard TR, Themann J, Stuempflen M, Peer AM, Vertesy A, Littleboy JB, Esk C, et al. Single-cell brain organoid screening identifies developmental defects in autism. Nature. 2023;621(7978):373–80. 37704762 10.1038/s41586-023-06473-y PMC10499611

[153] Lek A, Zhang Y, Woodman KG, Huang S, DeSimone AM, Cohen J, Ho V, Conner J, Mead L, Kodani A, et al. Applying genome-wide CRISPR-Cas9 screens for therapeutic discovery in facioscapulohumeral muscular dystrophy. Sci Transl Med. 2020;12(536):eaay0271. 32213627 10.1126/scitranslmed.aay0271 PMC7304480

[154] Torres VE, Harris PC. Autosomal dominant polycystic kidney disease: the last 3 years. Kidney Int. 2009;76(2):149–68. 19455193 10.1038/ki.2009.128 PMC2812475

[155] Batool L, Raab C, Beez CM, Kurtz A, Gollasch M, Rossbach B. Generation of human induced pluripotent stem cell line (BCRTi007-A) from urinary cells of a patient with autosomal dominant polycystic kidney disease. Stem Cell Res. 2023;69:103071. 36947994 10.1016/j.scr.2023.103071

[156] Linn AK, Maneepitasut W, Tubsuwan A, Kitiyanant N, Phakdeekitcharoen B, Borwornpinyo S, Hongeng S, Phanthong P. Establishment and characterization of MUi027-A: a novel patient-derived cell line of polycystic kidney disease with PKD1 mutation. J Pers Med. 2022;12(5):766. 35629189 10.3390/jpm12050766 PMC9145395

[157] Ma Y, Shang S, Shi M, Yang Y, Li Q, Bai XY. Establishment of the induced pluripotent stem cell line PLAFMCi006-A from peripheral blood mononuclear cells of polycystic kidney disease patients with PKD2 gene mutation. Stem Cell Res. 2022;60:102681. 35091308 10.1016/j.scr.2022.102681

[158] Drummond E, Wisniewski T. Alzheimer’s disease: experimental models and reality. Acta Neuropathol. 2017;133(2):155–75. 28025715 10.1007/s00401-016-1662-x PMC5253109

[159] Kuraoka S, Tanigawa S, Taguchi A, Hotta A, Nakazato H, Osafune K, Kobayashi A, Nishinakamura R. PKD1-dependent renal cystogenesis in human induced pluripotent stem cell-derived ureteric bud/collecting duct organoids. J Am Soc Nephrol. 2020;31(10):2355–71. 32747355 10.1681/ASN.2020030378 PMC7609014

[160] Shimizu T, Mae SI, Araoka T, Okita K, Hotta A, Yamagata K, Osafune K. A novel ADPKD model using kidney organoids derived from disease-specific human iPSCs. Biochem Biophys Res Commun. 2020;529(4):1186–94. 32819584 10.1016/j.bbrc.2020.06.141

[161] Natri HM, Del Azodi CB, Peter L, Taylor CJ, Chugh S, Kendle R, Chung MI, Flaherty DK, Matlock BK, Calvi CL, et al. Cell type-specific and disease-associated eQTL in the human lung. bioRxiv. 2023;37:109.

[162] Ilieva H, Vullaganti M, Kwan J. Advances in molecular pathology, diagnosis, and treatment of amyotrophic lateral sclerosis. BMJ. 2023;383:e075037. 37890889 10.1136/bmj-2023-075037 PMC10603569

[163] Kampmann M. Elucidating drug targets and mechanisms of action by genetic screens in mammalian cells. Chem Commun (Camb). 2017;53(53):7162–7. 28487920 10.1039/C7CC02349A PMC5507204

[164] West-Livingston LN, Park J, Lee SJ, Atala A, Yoo JJ. The role of the microenvironment in controlling the fate of bioprinted stem cells. Chem Rev. 2020;120(19):11056–92. 32558555 10.1021/acs.chemrev.0c00126 PMC7676498

[165] Khan T, Waseem R, Shahid M, Ansari J, Ahanger IA, Hassan I, Islam A. Recent advancement in therapeutic strategies for Alzheimer’s disease: insights from clinical trials. Ageing Res Rev. 2023;92:102113. 37918760 10.1016/j.arr.2023.102113

[166] Ortabozkoyun H, Huang PY, Cho H, Narendra V, LeRoy G, Gonzalez-Buendia E, Skok JA, Tsirigos A, Mazzoni EO, Reinberg D. CRISPR and biochemical screens identify MAZ as a cofactor in CTCF-mediated insulation at Hox clusters. Nat Genet. 2022;54(2):202–12. 35145304 10.1038/s41588-021-01008-5 PMC8837555

[167] Tian R, Abarientos A, Hong J, Hashemi SH, Yan R, Drager N, Leng K, Nalls MA, Singleton AB, Xu K, et al. Genome-wide CRISPRi/a screens in human neurons link lysosomal failure to ferroptosis. Nat Neurosci. 2021;24(7):1020–34. 34031600 10.1038/s41593-021-00862-0 PMC8254803

[168] Drager NM, Sattler SM, Huang CT, Teter OM, Leng K, Hashemi SH, Hong J, Aviles G, Clelland CD, Zhan L, et al. A CRISPRi/a platform in human iPSC-derived microglia uncovers regulators of disease states. Nat Neurosci. 2022;25(9):1149–62. 35953545 10.1038/s41593-022-01131-4 PMC9448678

[169] Samelson AJ, Ariqat N, McKetney J, Rohanitazangi G, Parra Bravo C, Goodness D, Tian R, Grosjean P, Abskharon R, Eisenberg D *et al*: CRISPR screens in iPSC-derived neurons reveal principles of tau proteostasis. *bioRxiv* 2023. 10.1016/j.cell.2025.12.038 PMC12978015 41610849

[170] Sanchez CG, Acker CM, Gray A, Varadarajan M, Song C, Cochran NR, Paula S, Lindeman A, An S, McAllister G, et al. Genome-wide CRISPR screen identifies protein pathways modulating tau protein levels in neurons. Commun Biol. 2021;4(1):736. 34127790 10.1038/s42003-021-02272-1 PMC8203616

[171] Cenik BK, Sze CC, Ryan CA, Das S, Cao K, Douillet D, Rendleman EJ, Zha D, Khan NH, Bartom E, et al. A synthetic lethality screen reveals ING5 as a genetic dependency of catalytically dead Set1A/COMPASS in mouse embryonic stem cells. Proc Natl Acad Sci USA. 2022;119(19):e2118385119. 35500115 10.1073/pnas.2118385119 PMC9171609

[172] Collier AJ, Bendall A, Fabian C, Malcolm AA, Tilgner K, Semprich CI, Wojdyla K, Nisi PS, Kishore K, Roamio Franklin VN, et al. Genome-wide screening identifies Polycomb repressive complex 1.3 as an essential regulator of human naive pluripotent cell reprogramming. Sci Adv. 2022;8(12):eabk0013. 35333572 10.1126/sciadv.abk0013 PMC8956265

[173] Sintov E, Nikolskiy I, Barrera V, Hyoje-Ryu Kenty J, Atkin AS, Gerace D, Ho Sui SJ, Boulanger K, Melton DA. Whole-genome CRISPR screening identifies genetic manipulations to reduce immune rejection of stem cell-derived islets. Stem Cell Reports. 2022;17(9):1976–90. 36055241 10.1016/j.stemcr.2022.08.002 PMC9481918

[174] Gao C, Qi X, Gao X, Li J, Qin Y, Yin Y, Gao F, Feng T, Wu S, Du X. A genome-wide CRISPR screen identifies factors regulating pluripotency exit in mouse embryonic stem cells. Cells. 2022;11(15):2289. 35892587 10.3390/cells11152289 PMC9331787

[175] Mamun MMA, Khan MR, Zhu Y, Zhang Y, Zhou S, Xu R, Bukhari I, Thorne RF, Li J, Zhang XD, et al. Stub1 maintains proteostasis of master transcription factors in embryonic stem cells. Cell Rep. 2022;39(10):110919. 35675767 10.1016/j.celrep.2022.110919

[176] Al Adhami H, Vallet J, Schaal C, Schumacher P, Bardet AF, Dumas M, Chicher J, Hammann P, Daujat S, Weber M. Systematic identification of factors involved in the silencing of germline genes in mouse embryonic stem cells. Nucl Acids Res. 2023;51(7):3130–49. 36772830 10.1093/nar/gkad071 PMC10123117

[177] Kaemena DF, Yoshihara M, Beniazza M, Ashmore J, Zhao S, Bertenstam M, Olariu V, Katayama S, Okita K, Tomlinson SR, et al. B1 SINE-binding ZFP266 impedes mouse iPSC generation through suppression of chromatin opening mediated by reprogramming factors. Nat Commun. 2023;14(1):488. 36717582 10.1038/s41467-023-36097-9 PMC9887000

[178] Li X, Yue X, Sepulveda H, Burt RA, Scott DA. S AC, S AM, Rao A: OGT controls mammalian cell viability by regulating the proteasome/mTOR/ mitochondrial axis. Proc Natl Acad Sci USA. 2023;120(3):e2218332120. 36626549 10.1073/pnas.2218332120 PMC9934350

